# EnABLe: An agent-based model to understand *Listeria* dynamics in food processing facilities

**DOI:** 10.1038/s41598-018-36654-z

**Published:** 2019-01-24

**Authors:** Claire Zoellner, Rachel Jennings, Martin Wiedmann, Renata Ivanek

**Affiliations:** 1 Department of Population Medicine and Diagnostic Sciences, Cornell University, Ithaca, NY 14853 USA; 2 Department of Food Science, Cornell University, Ithaca, NY 14853 USA

## Abstract

Detection of pathogens in food processing facilities by routine environmental monitoring (EM) is essential to reduce the risk of foodborne illness but is complicated by the complexity of equipment and environment surfaces. To optimize design of EM programs, we developed **EnABLe** (“Environmental monitoring with an Agent-Based Model of *Listeria*”), a detailed and customizable agent-based simulation of a built environment. **EnABLe** is presented here in a model system, tracing *Listeria* spp. (LS) (an indicator for conditions that allow the presence of the foodborne pathogen *Listeria monocytogenes*) on equipment and environment surfaces in a cold-smoked salmon facility. **EnABLe** was parameterized by existing literature and expert elicitation and validated with historical data. Simulations revealed different contamination dynamics and risks among equipment surfaces in terms of the presence, level and persistence of LS. Grouping of surfaces by their LS contamination dynamics identified connectivity and sanitary design as predictors of contamination, indicating that these features should be considered in the design of EM programs to detect LS. The **EnABLe** modeling approach is particularly timely for the frozen food industry, seeking science-based recommendations for EM, and may also be relevant to other complex environments where pathogen contamination presents risks for direct or indirect human exposure.

## Introduction

*Listeria monocytogenes* (LM) is an opportunistic foodborne pathogen that has been estimated to cause more than 23,000 illnesses and nearly 5,500 deaths worldwide each year^1^. Illness- and recall-associated costs to American consumers and food companies are estimated to exceed $4 billion annually^2^. LM can be present in a variety of environments and is typically associated with soiled and moist conditions, not uncommon in food production facilities^3–6^. Although the risk of foodborne illness due to LM can be reduced through heat treatment and/or the addition of antimicrobial ingredients or processes, numerous food products are exposed to the processing environment after heat treatment and may not undergo a cook step by the consumer prior to consumption (known as ready-to-eat or RTE, e.g., deli meats, ice cream, many cheeses, frozen fruits and vegetables used in smoothies) or are never exposed to lethal treatments during processing (e.g., fresh-cut produce and frozen fruit). Contaminated products have been traced back to events of environmental recontamination during processing^7,8^, thus highlighting the impact of LM in processing facilities on product safety and foodborne illness. Identification of LM sources in the facility via routine environmental monitoring (EM) is an essential practice to inform the corrective actions necessary for elimination of LM and prevention of recontamination.

EM involves routine collection of swab samples from equipment, tools, personnel, and the facility environment. While LM is the only human pathogen in the genus *Listeria*, environmental samples are often evaluated to detect the presence of *Listeria* spp. (LS) as a conservative approach to identifying conditions that will allow for LM presence. In general, previous experience in the facility and industry guidance are used, over statistically-based and random sampling approaches, when determining the number and location of samples taken for both routine EM programs and investigations into potential *Listeria* harborage sites^9–14^. Areas of a facility are often prioritized according to levels of required hygienic care (also known as hygienic zoning) and surfaces within each area may be designated into zones (also known as zoning) according to different levels of LM control and their proximity to food products, for example. These areas and surfaces are monitored with different frequencies or intensities to detect a loss of control and indicate the risk of product recontamination during processing^9,12^. The complexity of the processing equipment and environment, coupled with the heterogeneous distribution of contamination, present major challenges when designing EM programs and evaluating results. Despite the numerous publications on *Listeria* in food processing facilities, a recent extensive scoping review concluded that there is a need for individual facility-specific techniques to scientifically support alternative designs and implementation of EM programs^15^.

Simulation and risk assessment models are useful for EM decision-making, especially where experimental approaches would not be feasible or ethical and where empirical data are difficult to obtain. Quantitative risk assessment (QRA) models, employing discrete-event simulation, for management of LM during food processing and handling have previously considered processing equipment and environment compartments as potential sources of contamination of soft cheese and retail delicatessen products^16,17^. While these QRA models have improved understanding of LM cross-contamination and recontamination and the resulting consumer exposure to LM, both acknowledged gaps in modeling the growth and behavior of *Listeria* under specific environmental conditions and on equipment surfaces prior to contaminating food products. Agent-based modeling (ABM) is a complementary approach towards addressing this gap, sharing many features with a QRA (as it is also a simulation tool), that utilizes spatially explicit and rule-based computation to represent in detailed granularity the real-world components and day-to-day features of a system^18^, in order to preserve natural heterogeneity within and among facility environments and practices. The individuals, or agents, that make up an agent-based model are unique in their characteristics and autonomous in their interactions with each other and their environment^18^. Modeled continuously over time, the collective interactions of agents and their environment reveal both causes and consequences of emerging patterns. Thus, application of ABM in food processing facilities allows for simulation of microbial behavior under complex and dynamic environmental conditions and on surfaces that could not otherwise be modeled by analytically tractable mathematical models or experimented on using other methods.

Environmental monitoring with an Agent-Based Model of *Listeria* (**EnABLe**) is an *in silico* approach to simulate the behavior of LS in food processing environments in order to evaluate features and characteristics relevant for designing EM programs. **EnABLe** was implemented in this study to recreate the complex environment of a cold-smoked salmon processing facility and to model transmission of LS on the processing equipment and environmental surfaces. Cold-smoked salmon is an RTE food that does not undergo a heat treatment (“kill step”) or other listeriacidal treatment during processing and is exposed to the processing environment and equipment during slicing and packaging. The resulting *in silico* dataset of model parameter values and the corresponding presence and level of LS in the modeled and simulated cold-smoked salmon slicing room was used to evaluate risks of contamination within the processing equipment and environment. Of particular interest was identification of common features and characteristics of the environment that may contribute to LS (including LM) contamination and therefore should dictate the design of routine EM.

## Model and Methods

### Model approach and implementation

For purposes of demonstrating the approach and implementation, in this study **EnABLe** was applied to model the finished product slicing and packaging room, the high hygiene area, of a smoked salmon processing facility, in which the authors have previously conducted intensive *Listeria* sampling studies for over 7 years^19–22^. The production activities occurring in the slicing room are briefly explained in the following sentences to provide a context for the modeled environment, agents and simulation framework. Following the cold-smoking cycle, racks of salmon fillets are moved to a finished product cooler and held overnight. Product from this cooler enters the slicing room at the skinning area, where the flesh of the fillet is removed from the skin. Skinned fillets move through a window in the wall to the trimming area, where several employees use knives to trim belly fat, fins and the tail. Trimmed fillets are placed on one of four slicers to be thinly sliced and conveyed to the final staging and packaging area where employees hand portion, weigh and package the product. We used an estimate of 612 fillets processed per hour in an eight hour shift with three mandatory breaks; one half-hour and two 15-min breaks^21^. The facility operates on a single shift, 5 days a week, and has a designated cleaning crew that cleans and sanitizes the slicing room at the end of each daily shift^22^.

**Figure 1:**
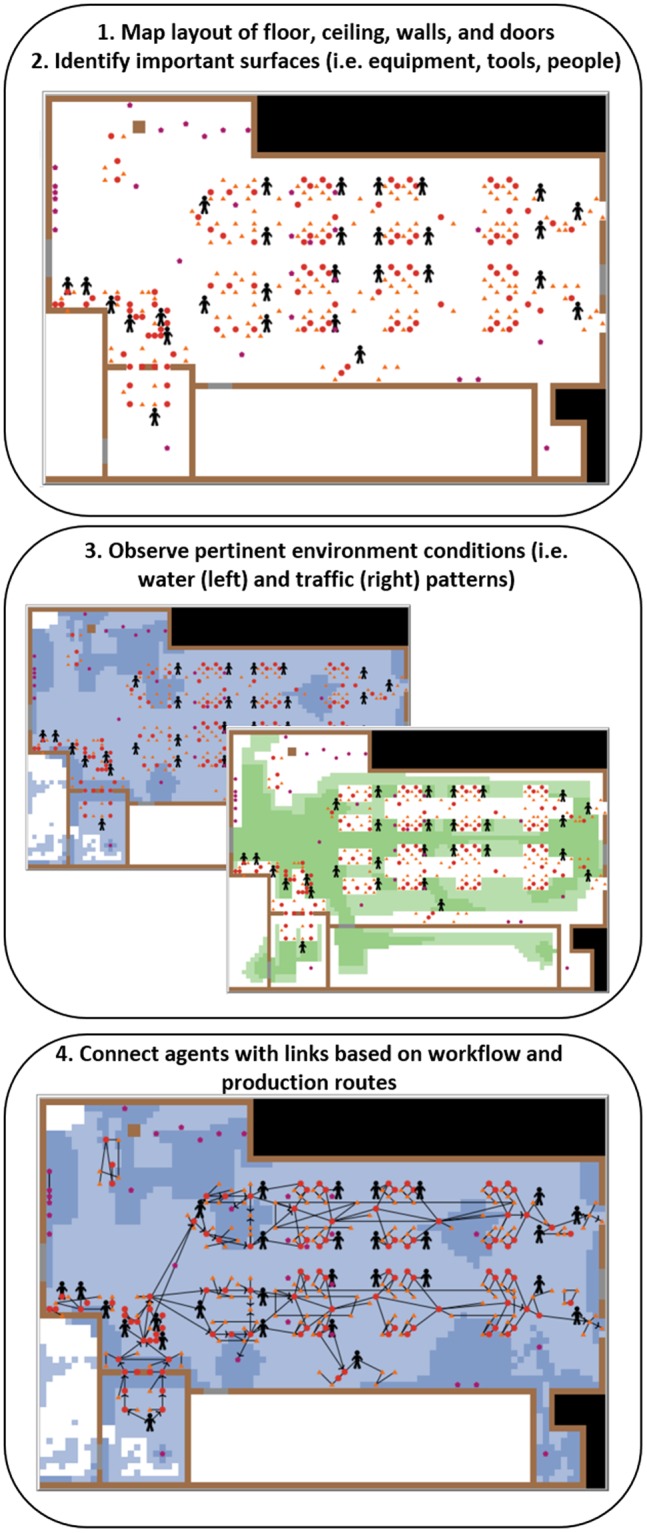
Method for **EnABLe** representation of *Listeria* spp. in cold-smoked salmon slicing room and equipment, implemented using NetLogo 6.0. *Step 1:* Observations were taken in the built environment so that the floors and ceiling (white), walls (brown), and doors (gray) could be represented in the model environment as 25 × 25 cm spaces. The black-shaded areas are outside the bounds of the modeled environment. *Step 2:* Items within the environment, such as equipment, tools and people, were modeled as a collection of agents representing the different food contact and non-contact surfaces. The shape and color of each agent signifies the zone category (red circles: Zone 1; orange triangles: Zone 2; purple pentagons: Zone 3; black stick figures: employees). *Step 3:* The presence of water, temperature of the room, and traffic patterns were mapped within the environment. Patches were colored by their water (blue) or traffic (green) state, depending on the view selected on the interface by the user. *Step 4:* Physical proximity and workflow were used to establish undirected and directed links, respectively, between agents, creating a network upon which *Listeria* spp. may spread.

The facility-specific model was developed in close collaboration with the facility personnel and integrated observed features, expert elicitation, and published data, when available, to define parameter values related to LS behavior in food processing environments: introduction, transmission, growth, and removal. An overall framework for the discretization procedure of the equipment, environment and pertinent facility-specific features is given in Fig. 1. Briefly, the Euclidean topology was applied to the floorplan of the slicing room, including the walls, floors and ceilings, as a grid of uniform squares, called patches (based on a 25 × 25 cm scale for each patch). Items within the slicing room environment, such as equipment, tools, and people, were represented as agents with defined spatial location, height, and characteristics further described below. Use of ABM topologies in the environment and agent height allowed for a semi-3D representation of the processing room.

Each piece of equipment in the slicing room environment (e.g., slicer) was represented in the model as a collection of agents comprising the different food contact and non-contact surfaces (e.g., control panel, in-belt, slicer blade), based on historical sampling sites, known and observed risk areas, and consultation with the food safety manager and related personnel. Conditions relevant to the presence of LS in the environment, specifically the presence of water and traffic patterns, were observed and overlaid upon the *in silico* discretized slicing room space. Physical proximity and workflow were used to establish links between agents, making a network through which LS may be transmitted. Directed links represented contamination routes via moving items, personnel, or physical contact and signified transmission of LS in one direction between two agents. Undirected (i.e., bidirectional) links represented contamination routes due to repeated physical contact or proximity and signified transmission of LS in both directions between two agents. The agents, links, and patches collectively formed the *in silico* model of the cold-smoked salmon slicing room in which LS contamination scenarios were visualized and evaluated.

**Figure 2:**
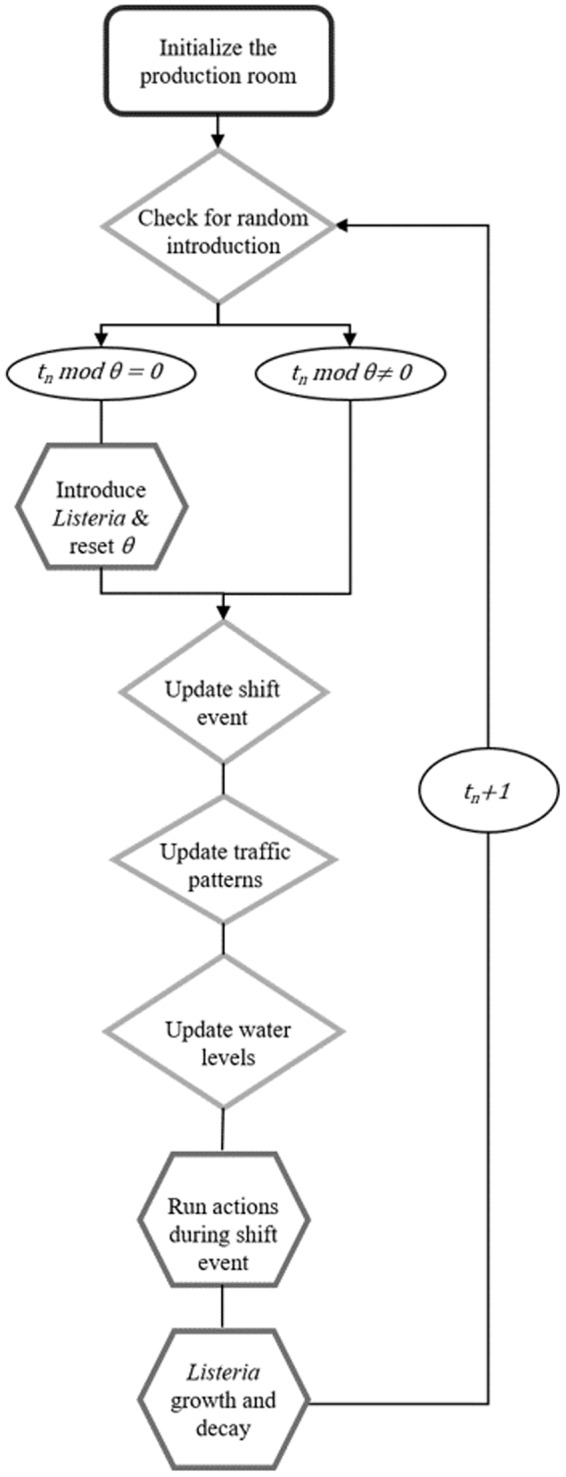
Flow diagram for hourly execution of **EnABLe**, where *t*_*n*_ is the current time (in hours) of the simulation and *θ* is the variable time at which *Listeria* spp. is introduced from outside the slicing room.

**EnABLe** was implemented using the open-source program NetLogo 6.0^23^ and the model is available upon request from the corresponding author. The authors chose to initialize the *in silico* processing plant upon setup of the simulation as 12:01AM on Sunday, at which time the cold-smoked salmon slicing room was empty. During setup the environment was divided into patches, the equipment and employee agents were created, and the initial parameter values were drawn from their respective probability distributions. Upon simulation, the model ticked through time in hours according to typical activities in the production schedule of the slicing room, checking for LS introduction, updating shift events and environmental conditions, executing sub-processes depending on the shift event, and allowing for LS growth and survival (Fig. 2).

### Model agents, inputs and sub-processes

**EnABLe** consisted of two agent types, equipment surfaces and employees; both were modeled as stationary objects but were able to interact with each other and the slicing room environment (patches). The majority of individual employee tasks in the cold-smoked slicing room could be assigned to a single station (approximately 1 m^2^) on the production line and were characterized by repetitive activities that move products on surfaces in close proximity to their station. Therefore, these activities were best modeled using a stationary agent as the employee with links connecting the employee to adjacent equipment and environment agents. Each individual agent had unique features, including surface area, proximity to food products (through classification into Zones 1 to 3; see below for more detailed descriptions), sanitary design (referred to in the model as cleanability), and connectivity to other agents and/or patches (defined by presence and type of links and spatial co-location). While there are different methods for zoning surfaces in a food processing facility, the U.S. Food and Drug Administration (FDA) approach was adopted in this model^12^. Zone 1 surfaces were those that directly contacted food products (also called food-contact surfaces, FCS). Zone 2 non-food-contact surfaces (NFCS) were those in close proximity to food and immediately surrounding FCS (e.g., equipment housing and framework). Zone 3 NFCS were those more distant from food and processing equipment (e.g., drains, trash bins, hoses, handwashing sink). As the model only applied to the equipment and environment within the slicing room, Zone 4 surfaces, which are defined as those surfaces located outside of the processing area but presenting a risk for introduction of LS into the processing environment (e.g., lockers, maintenance cart), were not included as agents. Instead, introduction of LS due to traffic from areas outside of but adjacent to the slicing room was included as a sub-process (Supplementary Information, Appendix I). Agents and patches had a binary descriptor of contamination (1 = contaminated, 0 = not contaminated), in addition to attributes such as the number of LS colonies (CFU) and concentration of LS on the surface (CFU/cm^2^), length of time contaminated, presence of water, and sampling history; these characteristics were dynamic and allowed to change over the simulated time. In total, there were 344 agents; summaries of agent attributes at set-up across zones are included in Table 1. The interactions between agents and patches due to hourly execution of activities during slicing room operation gave rise to the dynamic behavior of LS contamination in the built environment. The model was designed using sub-processes for introduction, growth/survival, transmission, removal, and EM, described in detail in the Supplementary Information (Appendix I), in order to be applicable to diverse production environments and schedules. Where possible, input parameter estimations came from several published papers, an existing *Listeria* risk model in retail delicatessens, and observations in the cold-smoked salmon facility (Table 2 and Table S1). Other parameters related to introduction and transmission of *Listeria* in food processing environments (Table 2 and Table S1) were estimated from an expert elicitation (Appendix II). Finally, several assumptions, explained and justified in the sub-process descriptions below, were necessary when literature and expert elicitation were not available.

**Table 1:** Summary of **EnABLe** agent characteristics at set-up by Zone.

	Zone 1^a^	Zone 2	Zone 3	Employees
Number of agents	133	166	45	29
Distance from floor (m)	0.9 [0.6, 1.2]^b^	0.9 [0.3, 1.2]	0.0 [0.0, 2.9]	1.2 [0.9, 1.2]
Surface area (cm^2^)	630 [70, 7500]	230 [25, 6300]	2500 [25, 7100]	160 [160, 160]
Number of out-directed links	0.0 [0.0, 2.0]	0.0 [0.0, 0.0]	0.0 [0.0, 0.0]	0.0 [0.0, 0.0]
Number of in-directed links	0.0 [0.0, 1.0]	0.0 [0.0, 0.0]	0.0 [0.0, 0.0]	0.0 [0.0, 0.0]
Number of undirected links	2.0 [1.0, 4.0]	2.0 [1.0, 3.0]	0.0 [0.0, 2.0]	1.0 [1.0, 3.0]
Number (%) not cleanable	9 (7%)	28 (17%)	32 (71%)	0 (0%)

^a^Zone 1 agents and the summary of their attributes include the employees in the rightmost column.

^b^Values given are median [5^th^–95^th^ percentile], unless otherwise stated.

**Table 2:** **EnABLe** input parameters, distribution information, values, and sources of information for *Listeria* spp. growth, reduction, introduction and floor transmission.

Symbol	Description^a^	Equation/Distribution	Mean	5^th^–95^th^ percentile	Reference
*p*_*z*_	Probability that *Listeria* spp. is introduced into the room via objects from Zone 4	10^Pert (−3.4, −2, −1.2, 4)^	0.01	[0.002, 0.03]	*expert opinion*
*N*_*z*_	Amount of *Listeria* spp. introduced per object from Zone 4 (CFU)	10^Pert (0, 0.7, 2, 4.6)^	10	[2, 27]	*expert opinion*
*R*_*d*_	Prevalence of *Listeria* spp. in cold-smoked salmon fillets on day *d*, for *d* = *Monday*, *Tuesday*, *Wednesday*, *Thursday*, *Friday*	10^Pert (−7, −4, −1, 4)^	10^−4^	[10^−5.9^, 10^−2.1^]	*expert opinion*
*N*_*R*_	Concentration of *Listeria* spp. per contaminated cold-smoked salmon fillet (CFU/g)	Gamma (1.2, 0.19)	6.3	[0.5, 18]	^41^
α	Proportion of *Listeria* spp. transferred to an equipment surface upon contact with a contaminated cold-smoked salmon fillet	10^Normal (−0.28, 0.2)^	0.56	[0.24, 1]	^50^
*p*_*r*_	Probability that a random event introduces *Listeria* spp. from outside the room	10^Pert (−3.4, −2, −1.2, 4)^	0.01	[0.002, 0.03]	*expert opinion*
*N*_*r*_	Amount of *Listeria* spp. introduced per random event (CFU)	10^Pert (0, 2.7, 4, 5)^	1862	[50, 6431]	*expert opinion*
*K*	Environmental carrying capacity of *Listeria* spp. (CFU/ml)	—	10^8^	—	^51^
GT	Generation time (h) of *Listeria* spp. on environment surfaces (10 °C)	Uniform (8.4, 24.4)	16.5	[9.2, 23.6]	^46, 47, 52^
μ	Maximum specific growth rate (h^−1^) of *Listeria* spp. on environment surfaces (10 °C)	=In (2)/GT	0.046	[0.03, 0.075]	^51^
*p*_*t*_	Probability that contact on floor from foot and equipment traffic is sufficient to spread *Listeria* spp. to adjacent patch	Pert (0.03, 0.25, 0.65, 4)	0.27	[0.10, 0.48]	^49^
*c*_*i*_	Contact rate between the contaminated patch and the adjacent patch given the traffic level *i = high*, *low*, *negligible*	*c*_*high*_ = 60/*patch*/*hr*, *c*_*low*_ = 12/*patch*/*hr*, *c*_*neg*_ = 0.2/*patch*/*hr*	—	—	*observed*
*p*_*w*_	Probability that environmental *Listeria* spp. is transported to adjacent patches via (visible) water	Uniform (0.01, 0.05)	0.03	[0.012, 0.048]	*assumed* ^*b*^
*β*	Transfer coefficient for *Listeria* spp. transmission among patches via traffic and water	Uniform (0.0, 0.05)	0.025	[0.002, 0.048]	*assumed*
*p*_*f*_	Probability that a cold-smoked salmon fillet falls to the floor during any given hour of production	Uniform (0.20, 0.40)	0.30	[0.20, 0.40]	*observed*
*p*_*c*_	Probability of a condensation transfer event given *Listeria* spp. is present	Uniform (0.01, 0.05)	0.03	[0.01, 0.05]	*assumed*
η_*d*_	Log_10_ reduction of *Listeria* spp. from washing and sanitation on day *d*, for *d*=*Monday*, *Tuesday*, *Wednesday*, *Thursday*, *Friday*	Pert (−8, −6, −1.5, 4)	−5.6	[−7.4, −3.5]	^17^
*γ*	Probability that a cleanable agent was not properly cleaned at the end of the shift	0.01	—	—	*assumed*

^a^All parameter values correspond to an hourly time-scale, the time-scale of the model.

^b^Assumptions were made when values were not available from literature or experts.

### Model verification and validation

Recognizing the importance of rigorous verification and validation of the developed model to assure its validity, we followed the definitions and approaches detailed by other ABM studies, particularly Codella *et al*.^24^. For model verification, agent logic was well-documented and the syntax was checked using NetLogo’s built-in debugger. Additional verification methods included visual testing and tracing of agents using the NetLogo interface, spot and stress tests using agent monitors, code reviews by peers, use of different seed generators, and inclusion of domain experts on *Listeria* in food processing environments throughout the process to confirm our logic and assumptions^18,24–26^.

For model validation, the simulations were designed to replicate historical sampling data of LS presence and absence on surfaces in the slicing room of the same cold-smoked salmon facility^22^ in order to validate that **EnABLe** accurately represented the model system. Simulated sampling as part of EM was conducted in the same manner as in a published study in the same cold-smoked salmon facility: Monday through Friday at the beginning, middle and end of production shift for 1 week on model agents across Zone 1 and Zone 2 surfaces^22^. This study setup was replicated in **EnABLe** using 10,000 numerical iterations, baseline parameter combinations, and a fixed random seed. LS prevalence was measured for the published outcomes across days of the week, time of production shift, area in the slicing room, and Zone 1 and Zone 2. In addition to graphical assessment, observed and predicted prevalence outcomes were compared using Chi-Square and Fischer’s Exact Test. Model simulations whose predicted daily prevalence fell within the 95% confidence interval (CI) of the observed daily prevalence were filtered and especially scrutinized to elucidate the details of the reality presented by the particular week in which the sampling study was conducted.

### Data and statistical analysis

As most input model parameters were represented with distributions describing the degree of variability surrounding the modeled process, NetLogo’s BehaviorSpace was used to run simulations with different combinations of parameter values. Parameters were intentionally defined as global variables in order to identify key drivers of contamination within the system. Therefore, global sensitivity analysis using partial rank correlation coefficients (PRCC) was conducted using the ‘epiR’ package, with bootstrap replicates done using the ‘sensitivity’ package, in R version 3.4.3 (from r-project.org)^27,28^. PRCCs provide a measure of monotonicity after the removal of the linear effects of all but one variable and were calculated to measure the strength of non-linear monotonic relationships between model inputs and different model outcomes to rank key model parameters driving LS contamination^29^. As the PRCC method is robust as long as correlation between the inputs is minimal or absent^29^, prior to PRCC analysis, parameters were confirmed to not be correlated and correction for multiple testing was applied using the Bonferroni approach^30^ (new significance threshold *P* = 0.05/53 = 0.0009). While validation simulations were focused on the subset of surfaces with available historical data, model outcomes and data from simulations were also collected for all agents and patches in the slicing room environment. Beyond validation, model outcomes were reported as median [5^th^–95^th^ percentile] unless otherwise stated. The differences in model outcomes among the agents were summarized by zone category (Zones 1–3) and were assessed graphically using violin plots and histograms and using repeated measures analysis of variance. Pairwise comparisons of mean LS prevalence (at the end of the modeled week) between zones were made using Chi-Square and Wilcoxon signed rank test. LS concentration at mid-shift and time spent contaminated per agent were log_10_-transformed and compared between zone categories using the Mann-Whitney U test. Again, correction for multiple testing was applied using the Bonferroni approach^30^ (new significance threshold *P* = 0.05/3 = 0.02). Cluster analysis (CA) was used to group agents into relatively homogeneous groups based on similarity of their attributes or their predicted contamination status. Due to the multidimensional dataset, factor and principle component analysis were first conducted to simplify the variables most important to describe slicing room agents^31^. The number of dimensions to use was determined using a cutoff of 80% cumulative proportion of variances retained. Hierarchical clustering was performed on the factor and principle component analysis results to join individuals into distinct clusters using Ward’s criterion^32^, which minimizes the sum of squared Euclidian distances between individuals within clusters while maximizing squared distance between the clusters, thus building a hierarchical tree. The number of clusters was initially determined by cutting the hierarchical tree and then improved upon through K-means clustering^33^. Principle component analysis, factor analysis and hierarchical clustering were conducted using the FactoMineR package^33^ in R version 3.4.3 (from r-project.org).

## Results

### Validation of **EnABLe** in cold-smoked salmon slicing room

**Figure 3:**
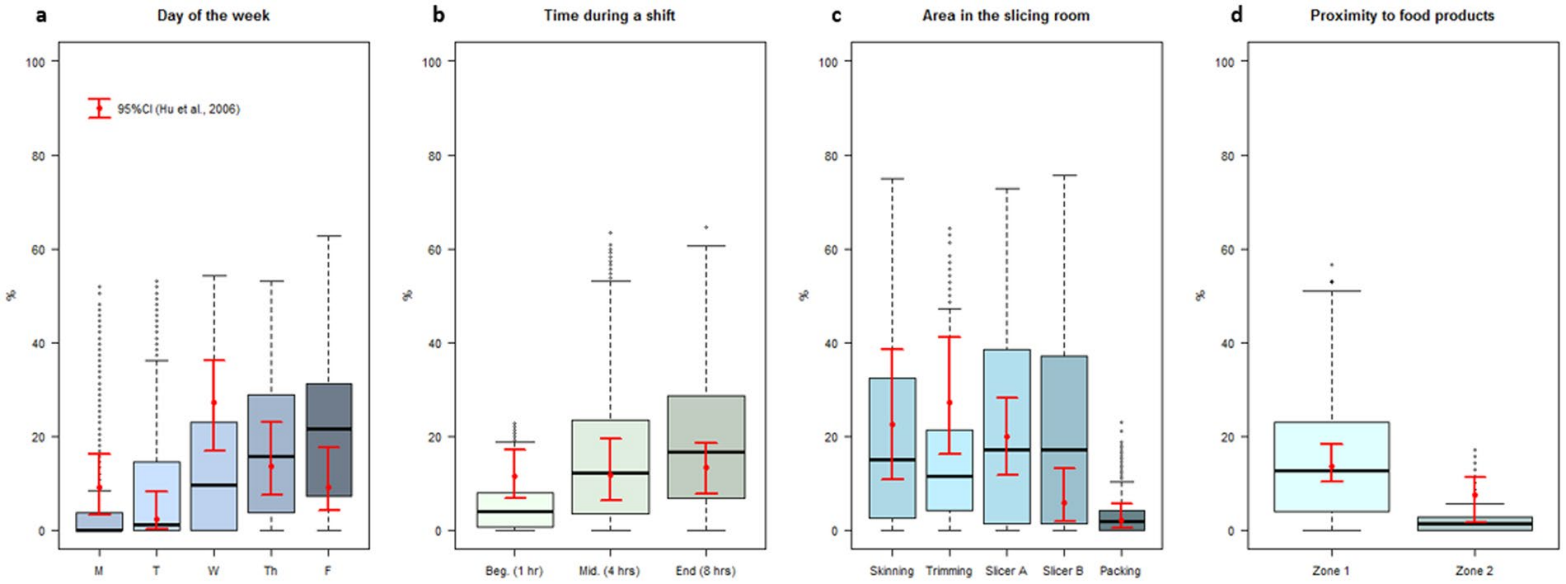
Validation of **EnABLe** with historical data. Boxplots show model simulation results as the median (black bar), interquartile range (box), 5^th^–95^th^ percentile (black whiskers), and outliers (black points outside of whiskers). The point and whiskers in red represent the observed prevalence and 95% confidence interval (CI)^22^. The model is compared to observed outcomes for *Listeria* spp. prevalence by (**a**) day of the week (Monday-Friday); (**b**) the time during a shift; (**c**) area of the slicing room; and (**d**) between Zone 1 and Zone 2 surfaces. Absence of significant differences between observed prevalence and mean simulated prevalence indicated model fit (Table S3).

The mean predicted prevalence of LS contamination on the fixed sites matching those sampled in Hu *et al*.^22^, (skinning area, trimming area, slicer A, slicer B, and packing area) varied between 2.7% and 21.7% (Table S3) and increased over day of the week and time during a production shift (Fig. 3a,b). LS contamination was focused in the skinning, trimming, and slicing areas and was higher on Zone 1 surfaces than Zone 2 surfaces (Fig. 3c,d). **EnABLe** simulations encompassed the 95% confidence intervals (CI) of the observed EM sampling results^22^ for LS prevalence Monday through Friday on fixed sampling sites across Zone 1 and Zone 2 surfaces at the beginning, middle, and end of production (Fig. 3). Comparison of the observed and modeled prevalence indicated no significant differences across the outcomes after Bonferroni correction (Table S3), although there was a spike in the observed LS prevalence on Wednesday that is not seen in the summarized simulation results in Fig. 3. We scrutinized our model iterations to identify what might have happened on Wednesday; this scenario illustrates an additional use of the model for the food industry, i.e., to investigate likely specific causes of certain contamination patterns in a given facility. Filtering of the model iterations using the observed 95% CI ranges during the study week confirmed the ability of the model to capture the particular circumstances present in that week (i.e., spike on Wednesday, followed by drop on Thursday and Friday), as a small proportion (0.1%) of the model iterations fell within the observed 95% CIs for all of the weekly prevalence outcomes. In these filtered iterations, prevalence of contaminated incoming fillets was higher on Monday and Wednesday and the concentration of LS in each fillet was higher compared to unfiltered iterations (data not shown). However, due to the highly stochastic nature of food processing (examples of sources of stochasticity include employee turnover, the source of raw materials, extreme weather events, and new equipment), that in the case of the available empirical validation data were unknown, during validation we chose not to calibrate the model parameters according to the filtered iterations that mimicked the spike on Wednesday and instead chose to emphasize the full range of LS contamination that may occur during production in the slicing room during any single week.

**Figure 4:**
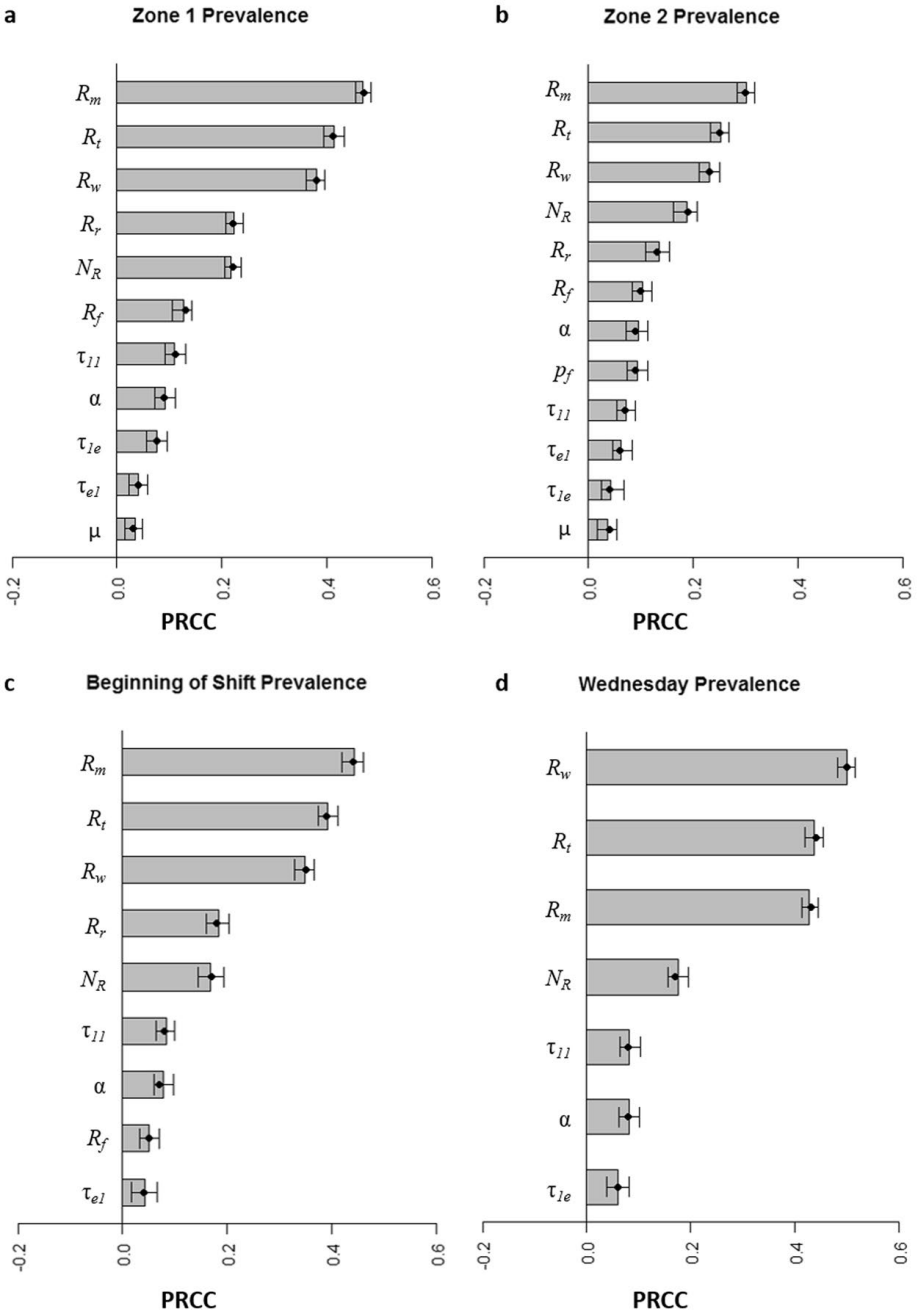
Key **EnABLe** parameters impacting *Listeria* spp. (LS) prevalence outcomes used in model validation: (**a**) Zone 1 surfaces, (**b**) Zone 2 surfaces, (**c**) At the beginning of the shift, and (**d**) on Wednesday. Tornado plots show Partial rank correlation coefficients (PRCC) and 95% confidence intervals for significant input parameters after Bonferroni correction. *R*_*m*_, prevalence of LS in cold-smoked salmon fillets on Monday; *R*_*t*_, prevalence of LS in cold-smoked salmon fillets on Tuesday; *R*_*w*_, prevalence of LS in cold-smoked salmon fillets on Wednesday; *R*_*r*_, prevalence of LS in cold-smoked salmon fillets on Thursday; *R*_*f*_, prevalence of LS in cold-smoked salmon fillets on Friday; *N*_*R*_, concentration (CFU/g) of LS per contaminated cold-smoked salmon fillet; α, proportion of LS transferred to an equipment surface upon contact with a contaminated cold-smoked salmon fillet; τ_11_, probability of LS transfer from Zone 1 to Zone 1 given contact; τ_1e_, probability of LS transfer from Zone 1 to an employee given contact; τ_e1_, probability of LS transfer from an employee to Zone 1 given contact; *p*_*f*_, probability that a cold-smoked salmon fillet falls to the floor during production; μ, growth rate (h^−1^) of LS on environment surfaces.

Subsequently, global sensitivity analysis was performed to identify key drivers of LS contamination within the model system. The key model parameters (interpreted by the rank of their correlation coefficient) influencing the variation of LS contamination on Zone 1 and Zone 2 surfaces, on Wednesday, and at the beginning of the shift were related to introduction of LS into the slicing room on the incoming smoked salmon fillets. For LS prevalence on Zone 1 (Fig. 4a), Zone 2 (Fig. 4b), beginning of shift (Fig. 4c) and slicer B (data not shown), the most influential parameter was fillet prevalence on Monday. For LS prevalence on Wednesday (Fig. 4d), the fillet prevalence on Wednesday was the most influential parameter. Both the concentration of LS in contaminated fillets (*N*_*R*_) and the proportion transferred to a surface (α) were positively correlated with LS prevalence (Fig. 4a–d). The proportion of LS transferred given different types of contact (e.g., τ_*11*_), probability of food falling (*p*_*f*_), and the growth rate (μ) ranked lower in importance on LS prevalence. Parameters for removal due to daily cleaning and sanitation were not significantly correlated with the LS prevalence outcomes after Bonferroni correction (*P* > 0.0009).

### LS contamination dynamics during production

**Figure 5:**
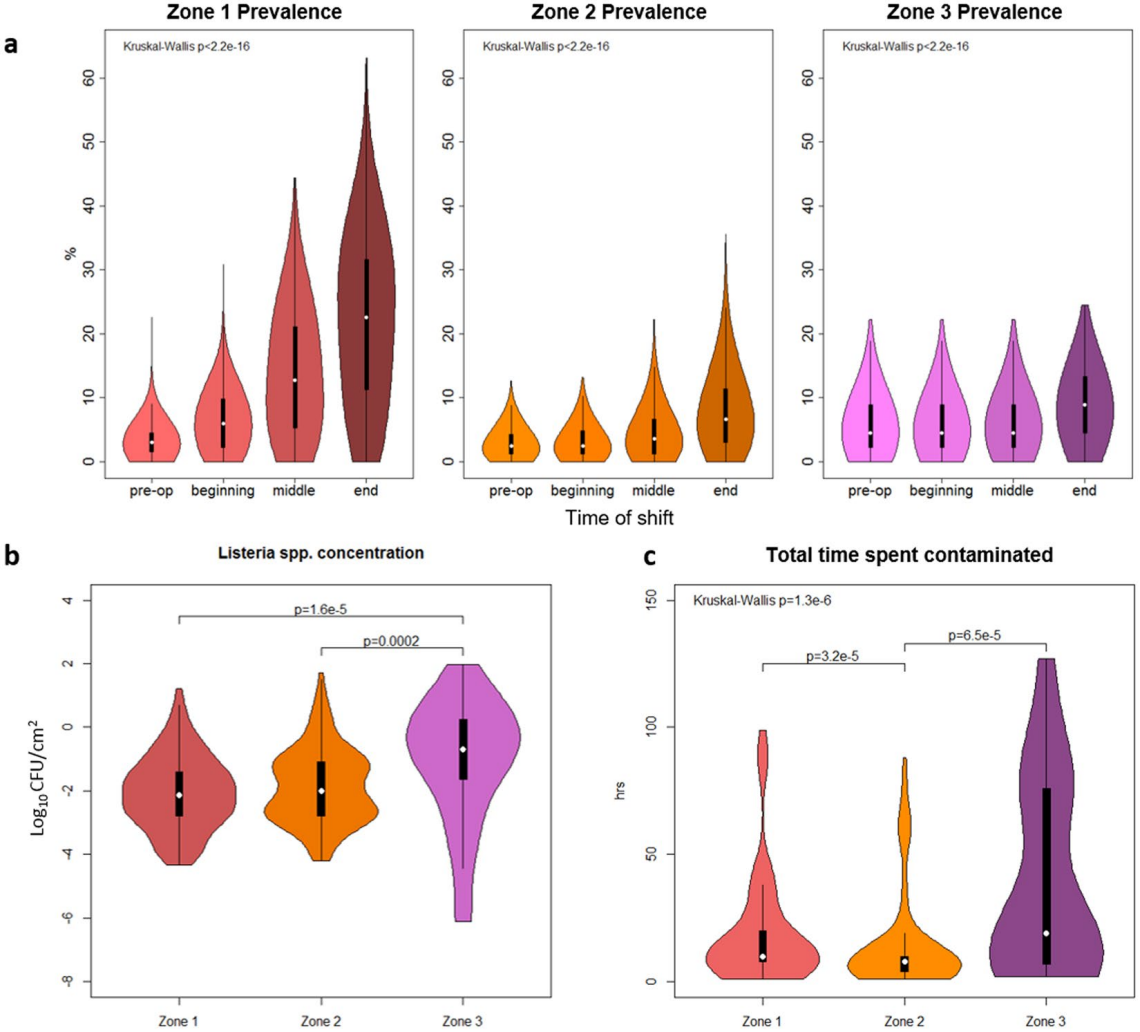
*Listeria* spp. (LS) dynamics on different surface types (characterized by their proximity to food products, with Zone 1 being in contact, and Zone 2 and Zone 3 being non-contact) in the cold-smoked salmon slicing room. (**a**) Simulation results for percent of sites contaminated over time of the shift on Friday are shown as violin plots, with the central white dot representing the median value, the black bar representing the interquartile range (IQR), the black line representing 95% confidence interval, and the outer shape representing the kernel density plot of all possible values (the thickest section indicates the mode). Mean LS prevalence differed significantly among slicing room surfaces from beginning to end of a production shift across all zone categories (*P* < 2.2e-16). (**b**) Simulation results for the concentration on Zone 1, 2, and 3 surfaces (Log_10_ CFU/cm^2^), if contaminated at the middle of the shift on Friday, shown as violin plots. The concentrations (described with median [5th and 95th percentile]) of LS on Zone 1 (−2.1 [−3.9, −0.05] Log_10_ CFU/cm^2^) and Zone 2 surfaces (−2.0 [−3.8, 0.15] Log_10_ CFU/cm^2^) were significantly different from Zone 3 (−0.7 [−5.7, 1.1] Log_10_ CFU/cm^2^) for this time point. (**c**) Violin plot of the total time (hours) spent contaminated by zone over one-week simulations. Total time contaminated described with median [5th and 95th percentile]) was significantly different across zone categories (*P* = 1.3e−6). Zone 1 and Zone 2 agents were contaminated for a cumulative of 10 hours [2.0, 87] and 8 hours [2.0, 62] over the simulated week, respectively, while Zone 3 sites were contaminated for a cumulative of 19 hrs [2.0, 113].

While the model validation was based on the equipment sites selected by Hu *et al*.^22^, the main value of **EnABLe** is the ability to evaluate new surfaces relevant for EM. Mean LS prevalence differed significantly among slicing room surfaces from beginning to end of a production shift for all three zone categories (Fig. 5a), with the greatest change between the beginning and end of the production shift occurring in Zone 1 ($\bar{{\Delta }}$ = 15.8 [0.75, 31.6]). At the end of the shift on Friday, mean prevalence of LS on Zone 1 surfaces was higher compared to Zone 2 (*P* = 0.0003), but was not significantly different from Zone 3 (*P* = 0.05), according to the Bonferroni corrected *P*-value. There was no difference in mean LS prevalence on Zone 2 and 3 surfaces at the end of the shift on Friday (*P* = 0.87). While Zone 1 surface contamination at the middle of a shift was most prevalent (compared to the other 2 zones), the concentration of LS on Zone 1 surfaces was not significantly different from Zone 2 and was significantly less than Zone 3 for this time point (Fig. 5b). Finally, the median amount of time (hours) an agent spent contaminated (both consecutively and interrupted) during the simulated week was significantly different across zone categories (Fig. 5c). The distribution of time contaminated by zone category was somewhat bimodal with the possibility that some surfaces may spend the majority of time contaminated, especially in Zone 3.

### Identification of similar surfaces among the processing equipment

CA was performed to explore grouping of agents according to their attributes or contamination status as a method to scientifically design an EM program. CA resulted in three clusters for both clustering methods (Table 3). Representative agents for each cluster were those with the shortest Euclidian distance from the center of the cluster and a complete list of the agents in each cluster is provided in the Supplementary Information.

**Table 3:** Groups of agents in the cold-smoked salmon slicing room identified by cluster analysis using either attributes or contamination outcomes over one week.

Cluster	Based on agent attributes (n = 344)			Based on agent contamination (n = 344)		
	A-I	A-II	A-III	C-I	C-II	C-III
Number of agents	40	264	40	8	299	37
*Zone 1*	0	97	36	1	97	35
*Zone 2*	0	162	4	3	163	0
*Zone 3*	40	5	0	4	39	2
Representative agent(s)	wall below hand sink	cart handle, cart handle, MBS control panel, slicer on-off switch, table underside	slicer in-belt	slicer gear joints	cutting table underside	cutting table top
**Cleanability**						
*Yes*	11	232	32	3	243	29
*No*	29	32	8	5	56	8
Distance from floor (m)^a^	0.21	0.93	0.85	0.42	0.85	0.84
**Area of slicing room**						
*Skinning*	2	8	7	0	8	9
*Trimming*	3	38	7	1	37	10
*Slicing*	6	28	14	1	31	12
*Packing*	10	156	5	2	167	2
*Mechanical Bone Separator (MBS)*	1	7	0	0	8	0
*Vacuum*	5	22	7	2	32	0
*Hand-washing*	4	2	0	1	5	0
*Ceiling*	0	3	0	0	3	0
*Cleaning*	9	0	0	1	8	0
Number of Out links^a^	0.0	0.0	1.1	0.0	0.02	0.97
Number of In links^a^	0.1	0.01	1.0	0.0	0.04	0.86
Number of Undirected links^a^	0.5	2.0	2.3	1.5	1.8	2.0
**Probability of LS contamination at mid-shift** ^a^						
*Monday*	0.01	0.01	0.05	<0.01	0.01	0.07
*Tuesday*	0.02	0.01	0.13	<0.01	0.01	0.17
*Wednesday*	0.04	0.02	0.22	<0.01	0.02	0.28
*Thursday*	0.06	0.03	0.29	<0.01	0.03	0.37
*Friday*	0.07	0.04	0.35	<0.01	0.04	0.45
**Concentration at mid-shift** ^a^ **(CFU/cm** ^**2**^ **)**						
*Monday*	1.3	0.50	0.25	4.9	0.46	0.39
*Tuesday*	1.2	0.21	0.12	5.8	0.18	0.14
*Wednesday*	1.9	0.36	0.12	12	0.26	0.14
*Thursday*	2.9	0.71	0.13	29	0.24	0.16
*Friday*	3.8	0.56	0.16	18	0.53	0.20
Contacts by contaminated agent^a^ (per wk, via link)	0.13	1.4	21	0.0	1.0	25
Transfers of contamination^a^ (per wk, via link)	0.0	0.37	26	0.0	0.17	30
Time contaminated^a^ (hrs)	36	14	38	47	16	43

^a^Mean of cluster.

Identifying clusters based on agent attributes, identified during model development, represented a practical way to assess contamination risk when little or no contamination data is available. Cluster A-I consisted of 40 agents in Zone 3, close to the floor (mean distance = 0.21 m), with no out-links and the majority not cleanable (73%). Cluster A-II consisted of 264 agents across Zone 1 (37%), 2 (61%), and 3 (2%), a mean distance from the floor of 0.93 m, with two undirected links, and the majority cleanable (88%). Cluster A-III consisted of 40 agents in Zone 1 (90%) and 2 (10%), a mean distance from the floor of 0.85 m, high connectivity, and the majority cleanable (80%). The predicted contamination across clusters A-I, A-II and A-III displayed three respective risk patterns: (i) rare contamination with relatively high LS concentrations; (ii) rare contamination with relatively low LS concentrations; and, (iii) frequent contamination with low LS concentrations.

Alternatively, analysis of aggregated **EnABLe** simulation data could be used to identify groups of agents (or sampling sites) that have similar contamination patterns. CA results based on agent contamination outcomes over one week identified three clusters and displayed the same three contamination patterns described above. Cluster C-I was comprised of 8 agents which were rarely contaminated (probability <0.01), but when contaminated they contained high concentrations at the middle of the shift (4.9–29 CFU/cm^2^). Cluster C-II was comprised of 299 agents, across Zone 1 (32%), 2 (55%), and 3 (13%), with low probability of contamination (0.01–0.04) and low levels of contamination (<1 CFU/cm^2^). Cluster C-III was comprised of 37 agents in Zone 1 (95%) and 3 (5%) that were frequently contaminated (probability = 0.07–0.45), but with low LS concentrations (<1 CFU/cm^2^). The agents in C-I were far more contaminated (in terms of concentration and length of time) and had little interaction with other agents (in terms of number of links) compared to agents in other clusters, suggesting that these sites were not successfully cleaned, allowed for growth, and were contaminated from patches, random events, or introduction from objects and areas outside of the slicing room via traffic.

## Discussion

In this study, we accomplished two main goals. First, we developed an *in silico* approach, using ABM, to recreate the environment of a food processing facility and simulate the behavior of LS during production. Second, we used the simulated LS behavior to assess contamination risks in the equipment and environment as relevant for designing EM programs. While the simulated LS dynamics are specific to the model system and cannot be used to extrapolate to all food processing facilities, **EnABLe** provided general findings for approaching EM in the diversity of food processing environments (e.g., frozen food facilities) or other complex built environments (e.g., health care facilities).

The **EnABLe** approach follows a research trend of using *in silico* models to design decision support tools and assess potential intervention strategies. Detection of pathogens in built environments, including hospitals, nursing homes and food processing facilities, by targeted or routine sampling is essential to reducing the risk of transmission of pathogens to humans, directly or indirectly (e.g., through foods), and the incidence of infection. ABM has been used previously to support design and implementation of infection control and management of contamination, for example in transmission of *Clostridium difficile* and spread of infections in healthcare settings^24,34,35^, and in evaluating surveillance protocols for detection of outbreaks of influenza in metropolitan areas^36,37^. ABM has also been used by national security officials and emergency managers to test response plans following catastrophic events, such as nuclear attacks^38^ and terrorist attacks on water distribution networks^39^. Similarly, food manufacturing companies and their food safety managers design EM programs in anticipation of rare but potentially devastating contamination events. Therefore, **EnABLe** detailed the cold-smoked salmon slicing room environment, equipment and conditions to thoroughly model the behavior and transmission of LS contamination over time. The specific benefit of the agent-based model is that unique features or changes in the modeled facility environment are reflected in the LS dynamics, thus providing a customized tool for decision-making. As with other decision support tools based on model simulations, the food industry should not expect **EnABLe** to make specific predictions about a particular event occurring. Instead, aggregate model simulations may be used to identify the likely range of contamination outcomes that result from current or hypothetical environment conditions and practices. Furthermore, ABM may be used as a tool to test the value of alternative actions^38^, such as alternative EM sampling approaches or capital investments in more sanitary equipment in the case of *Listeria* control. Through implementation in NetLogo, **EnABLe** can, and is intended to, be adapted to other facilities or environments (e.g., frozen food manufacturing) following the same approach (Fig. 1) and appropriate pathogen/product-specific parameterization.

In the **EnABLe** model of the slicing room, the daily prevalence of LS in the incoming cold-smoked salmon fillets was the main parameter influencing contamination on equipment. Cold-smoked salmon is an RTE food that does not undergo a heat treatment (“kill step”) or other listeriacidal treatment and therefore can be sporadically contaminated with LM^40^. **EnABLe** simulations elucidated that sporadic cold-smoked salmon contamination has an impact on LS in the processing room environment. In addition, the concentration of LS in each contaminated fillet ranked among the key parameters influencing Zone 1 prevalence, even though other studies cite *Listeria* contamination in fillets is most often detected at levels <100 CFU/g^41^. Introduction via random events and traffic from Zone 4 were not significant drivers of Zone 1 or 2 prevalence and have been previously recognized as minor risk factors compared to growth within niches^9^. Once on a Zone 1 surface, LS may be transferred to other equipment and food surfaces, may remain on the surface, or may be removed during routine cleaning and sanitation at the end of the shift^42^. Sensitivity analysis showed that LS prevalence in Zone 1 and Zone 2 was positively influenced by contact between Zone 1 surfaces and between Zone 1 and employees. Although these results were specific for this model system and may not be applicable for all RTE foods, the ability to identify key mechanisms of LS introduction and transmission within the processing environment provides actionable areas for eliminating LS sources and reducing the impact of contamination. Our results specifically suggest that improved control of LS on raw materials and in areas of frequent employee-to-food contact will be important for reducing not just product contamination, but also environmental contamination, which may be particularly relevant for other RTE products without a “kill step” (e.g., fresh produce and frozen fruit).

The model-predicted results for frequency, level and duration of LS contamination on equipment surfaces in the slicing room provide quantitative support for the design of EM programs. As previously described, zone classification is common practice for EM sample selection and several guidance documents recommend that food processing facilities sample both Zone 1 and Zone 2 surfaces at least 4 hours into production^12^. In addition, regulatory agencies and customer requirements increasingly demand scientifically supported EM programs; in particular, they suggest that the frequency of sampling, selection of sample locations and the number of sites tested be based on the risk of contamination with LM^12^. In an effort to provide more quantitative support for current EM practices, **EnABLe** simulations were focused on three main outcomes for sites in Zones 1–3: (i) the change in prevalence over the production shift and day of the week, (ii) the level of contamination per sampling site, and (iii) the amount of time a sampling site spends contaminated per week. The change in LS prevalence over the shift suggested that Zone 1 surfaces were more likely to test positive for LS after progression of production activities in the slicing room for several hours; this supports recommendations that collection of EM samples should occur several hours into production^12^. Our data also support the value of sampling Zone 1 surfaces, particularly in facilities where the food materials handled are initially contaminated^43^; we acknowledge though that Zone 1 sampling may not be able to easily differentiate between pathogens introduced from raw material and environmental sources, complicating interpretation of these data in practice. However, our conclusions need to be validated with models representing other facilities and foods. Although enumeration of LS-positive samples is not common practice, the level of contamination, along with prevalence, is relevant when modeling the likelihood of transmission and harborage in the processing facility environment. While Zone 1 sites had higher probabilities of being contaminated during production, the LS concentrations in both Zone 1 and Zone 2 were generally low compared to Zone 3, which may suggest lower risk for transferring contamination. However, connectivity should also be considered when evaluating the risk because a highly connected surface may widely transfer contamination even if contaminated at a low level at any given point in time^22^. Finally, the time spent contaminated is not generally used in assessing potential EM sites, but, together with prevalence and concentration data, may serve as a proxy in our model for identifying sites of intermittent versus more persistent contamination. For example, Zone 3 sites were found to generally contain higher concentrations and spend longer time contaminated, which provides information for identifying areas that require targeted sampling and/or sanitation. Furthermore, the frequency of sampling as part of EM can be optimized based on the time spent contaminated. Specifically, surfaces contaminated intermittently would require more frequent sampling to detect the contamination problem compared to surfaces that tend to be contaminated for longer stretches of time.

In addition to zoning and sanitary design, connectivity of surfaces in the slicing room environment contributed to contamination risks and should be considered when designing EM programs. It is widely recommended that EM programs be designed incorporating experience in the facility, historical data, and an aggressive approach to finding LS^9^. However, classification of a sampling site to a particular zone category is not always straightforward, even among experts, and usually depends on an understanding of its spatial context within the processing facility^43^. To evaluate all of these aforementioned features and characteristics of sampling sites relevant for improving the design of EM programs, LS contamination results from **EnABLe** simulations were summarized with CA. The sites that were highly connected (≥2 undirected links) and located upstream in the handling process were more likely to be contaminated when evaluated at the middle of the shift. Sites that were modeled as not cleanable did not appear in a single cluster but were overrepresented in clusters with high concentrations at the middle of the production shift and longer time spent contaminated. Systematic random sampling could be implemented using these clusters as subdivisions of the processing environment and then randomly selecting sites within each cluster.

Although the ABM approach accommodates high levels of complexity to achieve realism, some assumptions in parameter estimation and simplifications during model development were required and, thus, the current model may represent a “worst-case scenario” for or a limited prediction of LS transmission in the modeled environment. We assumed that patterns of water and traffic on the floor over time were the same each day because we observed production on a single day and lacked the data to support that they would differ day to day. Some sub-process parameter values (noted in Table 2, e.g., transmission of LS in visible water) were assumed because we lacked the data and information required for estimation. The amount of available empirical data from this facility was limited to one week, but if more extensive sampling over multiple weeks is available, more calibrated model parameters may be appropriate. At present, the model focuses on the short-term predictions of LS contamination risk by modeling many iterations of a single week in this simulated production facility, which we recognize would be a limitation if the goal is to design a long-term sampling plan. Long-term predictions would require also modeling of corrective actions after sampling identifies a positive site, which is expected to prevent accumulation of contamination over time. While inclusion of niches in the model (i.e., cleanability of surfaces) implicitly accounts for biofilms, in the future, the model could be further modified to more explicitly include biofilm formation and removal, which would require modeling of multiple bacterial states (e.g., cells in biofilm/not in biofilm). Lastly, we have developed a model- and simulation-based decision support tool according to current industry and regulatory practices for EM which are focused on LS in the environment. Our model did not estimate contamination in the final food product for three main reasons: (i) we modeled LS in the processing environment as an indicator because of its higher prevalence compared to LM, however LS in food products does not necessarily present a public health risk and is therefore not tested in final products; (ii) there was not sufficient data for LS in finished product leaving the slicing room to validate the model; (iii) presentation of data linked to product contamination may limit industry adoption of this model because of the perceived legal and regulatory liability. The current version of the model, though particularly focused on LS in the processing environment, may be used as a basis to develop LM-specific models in the future.

In conclusion, simulation models of *Listeria* transmission in the environment provide helpful decision support tools to design and assess EM strategies in a variety of food processing environments (e.g., frozen, RTE seafood, fresh produce). **EnABLe** simulation of LS dynamics in food processing facility environments should complement industry-wide efforts to optimize and validate the design of EM and *Listeria* control strategies and to improve communication of and adherence to sanitation standard operating procedures. We do not aim to predict the exact values of LS prevalence and concentration, but rather to elucidate differential risks for contamination and transmission within the processing equipment and environment. Combined with data analytics, the results of **EnABLe** can support risk-based decision making and management of EM programs. Specifically, CA revealed groups of similar surfaces with distinct contamination patterns and provided a starting point for identifying new sampling sites based on connectivity, sanitary design, traffic, and product flow. Future related applications include: (i) comparison of different sampling approaches for their ability to detect LS contamination; (ii) quantifying the effect of different interventions on environmental LS contamination patterns (i.e., to assess redesign of equipment from uncleanable to cleanable); and (iii) optimizing the number and connectivity of agents in different sized facilities to reduce LS transmission while maintaining productivity. This framework is particularly timely as data analytics are introduced to food safety and may also find application more broadly to other built environments.

## Electronic supplementary material

Supplemental Materials

## Data Availability

The **EnABLe** model developed during the current study and relevant files for simulation are available from the corresponding author on reasonable request.

## References

[1] de Noordhout CM (2014). The global burden of listeriosis: a systematic review and meta-analysis. Lancet Infect. Dis..

[2] Ivanek R, GröHn YT, Tauer LW, Wiedmann M (2005). The cost and benefit of *Listeria monocytogenes* food safety measures. Crit. Rev. Food Sci. Nutr..

[3] Ferreira V, Wiedmann M, Teixeira P, Stasiewicz MJ (2014). *Listeria monocytogenes* persistence in food-associated environments: epidemiology, strain characteristics, and implications for public health. J Food Prot.

[4] Sauders BD (2006). Molecular characterization of *Listeria monocytogenes* from natural and urban environments. J. Food Prot..

[5] Sauders BD (2012). Diversity of *Listeria* species in urban and natural environments. Appl. Environ. Microbiol..

[6] Carpentier B, Cerf O (2011). Review–Persistence of *Listeria monocytogenes* in food industry equipment and premises. Int. J. Food Microbiol..

[7] Currie A (2015). Multi-province listeriosis outbreak linked to contaminated deli meat consumed primarily in institutional settings, Canada, 2008. Foodborne Pathog. Dis..

[8] Laksanalamai P (2012). Genomic characterization of *Listeria monocytogenes* strains involved in a multistate listeriosis outbreak associated with cantaloupe in US. PLoS ONE.

[9] Tompkin RB (2002). Control of *Listeria monocytogenes* in the food-processing environment. J. Food Prot..

[10] Butts, J. Seek & destroy: Identifying and controlling *Listeria monocytogene*s growth niches. *Food Safety Magazin*e (2003). Available at: https://www.foodsafetymagazine.com/magazine-archive1/aprilmay-2003/seek-destroy-identifying-and-controlling-listeria-monocytogenes-growth-niches/. (Accessed on: 5th November 2018).

[11] Malley TJV, Butts J, Wiedmann M (2015). Seek and destroy process: *Listeria monocytogenes* process controls in the ready-to-eat meat and poultry industry. J. Food Prot..

[12] FDA-CFSAN. Control of *Listeria monocytogene*s in Ready-to-Eat Foods: Guidance for Industry. Draft Guidance. (Food and Drug Administration, 2017). Available at, http://www.fda.gov/downloads/Food/GuidanceRegulation/GuidanceDocumentsRegulatoryInformation/UCM535981.pdf#page=1&zoom=auto,-121,792 (Accessed on: 29th June 2018).

[13] International Dairy Foods Association. *Listeria* Control Resources for the Ice Cream and Frozen Ready-to-Eat Dairy-Based Dessert Industry. (International DairyFoods Association, 2016). Available at, http://www.idfa.org/docs/default-source/d-news/idfa-iica-listeria-control-resource-guide-051316.pdf (Accessed on: 29th June 2018).

[14] United Fresh Produce Association. Guidance on environmental monitoring and control of *Listeri*a for the fresh produce industry. (United Fresh Produce Association, 2013). Available at, http://www.centerforproducesafety.org/amass/documents/document/263/Listeria%20Guidance%20UFPA%202013.pdf (Accessed on: 11th May 2018).

[15] Zoellner C, Ceres K, Ghezzi-Kopel K, Wiedmann M, Ivanek R (2018). Design elements of *Listeria* environmental monitoring programs in food processing facilities: A scoping review of research and guidance materials. Compr. Rev. Food Sci. Food Saf..

[16] Tenenhaus-Aziza F, Daudin JJ, Maffre A, Sanaa M (2014). Risk-based approach for microbiological food safety management in the dairy industry: the case of *Listeria monocytogenes* in soft cheese made from pasteurized milk. Risk Anal..

[17] FDA/FSIS. Interagency Risk Assessment: *Listeria monocytogenes* in Retail Delicatessens; Technical Report. 1–175 (Food and Drug Administration, United States Department of Agriculture, 2013). Available at, https://www.fsis.usda.gov/wps/wcm/connect/c0c6dfbc-ad83-47c1-bcb8-8db6583f762b/Lm-Retail-Technical-Report.pdf?MOD=AJPERES (Accessed on: 26^th^ June 2018).

[18] Railsback, S. F. & Grimm, V. *Agent-based and individual-based modeling: a practical introduction*. (Princeton University Press, 2012).

[19] Thimothe J, Nightingale KK, Gall K, Scott VN, Wiedmann M (2004). Tracking of *Listeria monocytogenes* in smoked fish processing plants. J. Food Prot..

[20] Lappi VR (2004). Longitudinal studies on *Listeria* in smoked fish plants: impact of intervention strategies on contamination patterns. J. Food Prot..

[21] Ivanek R, Grohn YT, Wiedmann M, Wells MT (2004). Mathematical model of *Listeria monocytogenes* cross-contamination in a fish processing plant. J. Food Prot..

[22] Hu Y (2006). Daily variability of *Listeria* contamination patterns in a cold-smoked salmon processing operation. J. Food Prot..

[23] Wilensky, U. *NetLogo*. (Center for Connected Learning and Computer-Based Modeling, Northwestern University, 1999).

[24] Codella J, Safdar N, Heffernan R, Alagoz O (2015). An agent-based simulation model for *Clostridium difficile* infection control. Med. Decis. Making.

[25] Kopec JA (2010). Validation of population-based disease simulation models: a review of concepts and methods. BMC Public Health.

[26] Xiang, X., Kennedy, R., Madey, G. & Cabaniss, S. Verification and validation of agent-based scientific simulation models. In *Proceedings of the 2005 Agent-Directed Simulation Symposium* 37, 47–55 (The Society for Modeling and Simulation International, 2005).

[27] Stevenson, M. Package ‘epiR’: Tools for the Analysis of Epidemiological Data. Available at, https://cran.r-project.org/web/packages/epiR/epiR.pdf (Accessed on: 5^th^ November 2018) (CRAN, 2018).

[28] Iooss, B., Janon, A. & Pujol, G. Package ‘sensitivity’: Global Sensitivity Analysis of Model Outputs. Available at, https://cran.r-project.org/web/packages/sensitivity/sensitivity.pdf (Accessed on: 5^th^ November 2018) (CRAN, 2018).

[29] Marino S, Hogue IB, Ray CJ, Kirschner DE (2008). A methodology for performing global uncertainty and sensitivity analysis in systems biology. J. Theor. Biol..

[30] Dohoo, I., Martin, W. & Stryhn, H. *Veterinary epidemiologic research*. (VER Inc, 2010).

[31] Kassambara, A. HCPC - Hierarchical Clustering on Principal Components: Essentials. *STHDA - Statistical tools for high-throughput data analysi*s Available at, http://www.sthda.com/english/articles/31-principal-component-methods-in-r-practical-guide/117-hcpc-hierarchical-clustering-on-principal-components-essentials/ (Accessed: 26th June 2018) (2017).

[32] Ward JH (1963). Hierarchical grouping to optimize an objective function. J. Am. Stat. Assoc..

[33] Husson, F., Josse, J., Le, S. & Mazet, J. Package ‘FactoMineR’ (V 1.41): Multivariate exploratory data analysis and data mining. Available at: https://cran.r-project.org/web/packages/FactoMineR/FactoMineR.pdf (Accessed on: 5^th^ November 2018) (CRAN, 2018).

[34] Bintz J, Lenhart S, Lanzas C (2017). Antimicrobial stewardship and environmental decontamination for the control of *Clostridium difficile* transmission in healthcare settings. Bull. Math. Biol..

[35] Rubin MA (2013). A simulation-based assessment of strategies to control *Clostridium difficile* transmission and infection. PLoS ONE.

[36] Lewis, B., Eubank, S., Abrams, A. M. & Kleinman, K. *In silico* surveillance: evaluating outbreak detection with simulation models. *BMC Med*. *Inform*. *Decis*. *Mak*. **13**, 10.1186/1472-6947-13-12 (2013). 10.1186/1472-6947-13-12 PMC3691709 23343523

[37] Zhao, Y., Mei, S. & Zhang, W. Irregular spatial cluster detection based on H1N1 Flu simulation in Beijing. In *Modeling*, *Design and Simulation of Systems* 169–179, 10.1007/978-981-10-6463-0_15 (Springer, Singapore, 2017).

[38] Waldrop MM (2018). Free agents. Science.

[39] Monroe J, Ramsey E, Berglund E (2018). Allocating countermeasures to defend water distribution systems against terrorist attack. Reliab. Eng. Syst. Saf..

[40] Rorvik LM (2000). List*er*ia *mo*nocytoge*ne*s in the smoked salmon industry. Int. J. Food Microbiol..

[41] Eklund MW (1995). Incidence and sources of *Listeria monocytogenes* in cold-smoked fishery products and processing plants. J. Food Prot..

[42] Gallagher, D. L., Ebel, E. D. & Kause, J. R. *FSIS Risk Assessment for Listeria monocytogenes in Deli Meat*s. (United States Department ofAgriculture, Food Safety and Inspection Service, 2003). Available at, https://www.fsis.usda.gov/shared/PDF/Lm_Deli_Risk_Assess_Final_2003.pdf (Accessed on: 25th June 2018).

[43] Simmons CK, Wiedmann M (2018). Identification and classification of sampling sites for pathogen environmental monitoring programs for *Listeria monocytogenes*: Results from an expert elicitation. Food Microbiol..

[44] Wen J, Karthikeyan S, Hawkins J, Anantheswaran RC, Knabel SJ (2013). *Listeria monocytogenes* responds to cell density as it transitions to the long-term-survival phase. Int. J. Food Microbiol..

[45] Wiedmann M, Wang S, Post L, Nightingale K (2014). Assessment criteria and approaches for rapid detection methods to be used in the food industry. J. Food Prot..

[46] Petran RL, Zottola EA (1989). A study of factors affecting growth and recovery of *Listeria monocytogenes* Scott A. J. Food Sci..

[47] FDA/FSIS. *Quantitative Assessment of Relative Risk to Public Health from Foodborne Listeria monocytogenes Among Selected Categories of Ready-to-Eat Foods*. (Food and Drug Administration, United States Department ofAgriculture, 2003). Available at, https://www.fda.gov/downloads/food/foodscienceresearch/ucm197330.pdf (Accessed: 26th June 2018).

[48] Vogel BF, Hansen LT, Mordhorst H, Gram L (2010). The survival of *Listeria monocytogenes* during long term desiccation is facilitated by sodium chloride and organic material. Int. J. Food Microbiol..

[49] Chambers MK, Ford MR, White DM, Barnes DL, Schiewer S (2009). Transport of fecal bacteria by boots and vehicle tires in a rural Alaskan community. J. Environ. Manage..

[50] Hoelzer K (2012). Estimation of *Listeria monocytogenes* transfer coefficients and efficacy of bacterial removal through cleaning and sanitation. Int. J. Food Microbiol..

[51] Giménez B, Dalgaard P (2004). Modelling and predicting the simultaneous growth of *Listeria monocytogenes* and spoilage micro-organisms in cold-smoked salmon. J. Appl. Microbiol..

[52] Begot C, Lebert I, Lebert A (1997). Variability of the response of 66 *Listeria monocytogenes* and *Listeria innocua* strains to different growth conditions. Food Microbiol..

