# Supplementary material for: EnABLe: An agent-based model to understand Listeria dynamics in food processing facilities
Source: Sci Rep. 2019 Jan 24;9:495. doi: 10.1038/s41598-018-36654-z (PMC6346090; doi:10.1038/s41598-018-36654-z)
Supplement: Supplementary file 1 — Supplemental Materials [file 41598_2018_36654_MOESM1_ESM.pdf]

Supplementary Information

Title:
EnABLe: An agent-based model to understand *Listeria* dynamics in food processing facilities

Authors:
Claire Zoellner[1]*, Rachel Jennings[1], Martin Wiedmann[2], Renata Ivanek[1]

Author affiliations:
[1]Department of Population Medicine and Diagnostic Sciences, [2]Department of Food Science, Cornell University, Ithaca NY 14853 USA

***Contact information for corresponding author:** cez23@cornell.edu

APPENDIX I

1. Introduction sub-processes

LS was introduced into the finished product processing room via three mechanisms: (i) carried into the room from rooms adjacent to the slicing room (Zone 4), during times of high traffic into the room (p_z=10^Pert[-3.4,-2,-1.2,4]; expert opinion); (ii) introduced from food materials entering the room each day, i.e., cold-smoked salmon fillets being skinned, sliced and packaged (R_d=10^Pert[-7,-4,-1,4]; expert opinion); and (iii) introduced by unpredictable and undefined random events, which followed a Poisson process with the time to the next event assumed to follow an exponential distribution with mean p_r=10^Pert[-3.4,-2,-1.2,4] (expert opinion). Introduction from Zone 4 was restricted to floor patches and agents in proximity to doorways and was modeled by adding a LS load (N_z=10^Pert[0, 0.7, 2.0, 4.6] CFU; expert opinion) to the patch or agent and updating its concentration. Introduction from random events was possible at any patch (floor or ceiling) or agent in the slicing room and was modeled by adding a LS load (N_r=10^Pert[0, 0.7, 4.0, 5] CFU; expert opinion) to the patch or agent and updating its concentration. Finally, introduction from an incoming cold-smoked salmon fillet (100 g) to be processed was limited to a Zone 1 surface in the skinning, trimming or slicing area and was dependent on the concentration of LS (N_R~Gamma[1.2, 0.19] CFU/g)[1] and the transfer coefficient (α=10^Normal[-0.28,0.2])[2]. The model considered only these three modes of introduction which were identified and estimated through expert elicitation (described in Appendix II). These introduction modes expanded upon previous models that generally considered the presence of some reservoir in the processing facility as the source of LS capable of contaminating a food contact surface. It is recognized that there may be other potential modes of introduction, however they were not considered in this model.

2. Growth and survival sub-process

In the modeled environment, growth only applied to LS on patches and agents containing either moisture or visible water (and assumed presence of a sufficient amount of nutrients from residual food) at a particular time (i.e., during the current tick).Growth was modeled hourly according to a solution of the primary Verhulst logistic function, which describes growth as proportional to the present population and the available nutrients[3]:

$$N_{t+1} = \frac{KN_t e^{\mu}}{K + N_t(e^{\mu} - 1)} \tag{1}$$

where N_t was the initial population in 1 cm of moisture on the surface at time t, CFU/cm^3, K was the carrying capacity of the environment, 10^8 CFU/cm^3 [4], and μ was the maximum specific growth rate, h^{-1}, generated for each model iteration according to:

$$\mu = ln(2)/GT \tag{2}$$

where the generation time (GT, h) was uniformly distributed within the range from 8.4 to 24.2 (GT~Uniform[8.4, 24.2]) for 10°C (pH=5.6 and a_w=1.0)[5–7]; the modeled room maintains temperature at 10°C. This equation and growth rate matched the common practice of modeling microbial growth in foods and was thus considered as reasonable. In the modeled environment, this growth only applied to LS on patches and agents containing either moisture or visible water (and assumed presence of a sufficient amount of nutrients from residual food) at a particular time (i.e., during a particular simulated hour). LS did not grow in dry areas, but did survive; that is, in our model the LS population experienced no net change in the absence of water. *Listeria* has been shown to survive desiccation for extended periods of time in food processing environments and similar conditions[8,9]. It was assumed that there was no lag time and the physiological state of

the cells (i.e., stressed, starved, exposed to sanitizer) was not considered to affect the growth rate. These assumptions were reasonable and necessary as sufficient data to model lag phase or to include physiological state of LS on equipment surfaces were not available. Furthermore, the model did not consider formation of *Listeria* spp. biofilms on surfaces or attachments to equipment that become increasingly stronger with time or increasingly resistant to disinfectants. It was assumed that cells are uniformly distributed on contaminated surfaces. These assumptions were made due to the high degree of uncertainty in our understanding of the involved processes and required model parameters and may present a model limitation. Several studies have concluded that persistent and presumed nonpersistent LM strains were equally susceptible to disinfectants[8,10]. The complexity of different biofilm growth rates and adhesion strengths has been acknowledged but excluded in a previous risk assessment model[11]. In other models, growth was not considered to occur on equipment surfaces at all[12,13]. Our use of site characteristics (i.e., cleanability and presence of water) and growth accounts for the possibility that *Listeria* persists on modeled surfaces and the environment.

3. Transmission sub-processes

LS transmission was modeled throughout the environmental patches and equipment according to hourly activities in the slicing room. Transmission occurred among patches, among agents, and between patches and agents, as described in the following sections.

3.1 Transmission among patches

LS was spread across the floor patches by foot traffic or within contiguous puddles of water. The presence of water was described as either absent (dry), moist, or visibly wet and was represented by a numerical code 0, 1, or 2, respectively, for each patch. Similarly, traffic patterns on the floor were observed and areas were classified as high, low, and negligible traffic. The

traffic levels were assigned contact rates of 60 contacts/hr, 12 contacts/hr and 0.2 contacts/hr, respectively, based on observations. The water and traffic states of each patch were dynamic over the production shift depending on the activity in the slicing room each hour. For example, during cleaning, when water was observed to be used to spray down equipment prior to use of detergents, all agents and patches in the model were updated to be "visibly wet." Similarly, traffic was higher at the beginning and end of a shift compared to the middle of a production shift.

The probability of LS transmission (p_{tj}) via foot traffic at time *t* and patch *j* was based on the incidence rate (r_j) of adjacent patches becoming contaminated from patch *j* due to the frequency of contacts associated with the traffic assigned to the patch *j* and its adjacent patches. This process was modeled as:

$$p_{tj} = 1 - e^{-r_j} \tag{3}$$

$$r_j = (P)(p_{1j})(c_i)(p_t) \tag{4}$$

where P was the probability that patch j was selected from all patches containing traffic ($P = 1/4593 = 0.0002$); p_{1j} was the fraction of patches adjacent to patch *j* that were in the same or higher traffic level; c_i was the contact rate between the contaminated patch *j* and the adjacent patch given the traffic level i at the contaminated patch *j* and was based on observations in the modeled room of the smoked-salmon facility of $c_{high} = 60/patch/hr$, $c_{low} = 12/patch/hr$, and $c_{neg} = 0.2/patch/hr$; p_t was the probability that contact was sufficient for LS transmission and was set to follow a Pert distribution (p_t~Pert[0.03, 0.25, 0.65, 4]), encompassing 95% confidence levels from one study reporting transfer of generic *Escherichia coli* from boots to a linoleum floor[14]. An example calculation of r_j for a contaminated patch *j* that is characterized by

high traffic and for which 5 out of 8 adjacent patches also have high traffic, with the probability that contact is sufficient for transmission is set at 0.05 would be: $r_j = (0.0002)(5/8)(60/patch/hr)(0.05) = 0.0004/patch/hr$. The probability of transmission from that contaminated patch to an adjacent patch in an hour would then be: $p_{tj} = 1 - e^{-0.0004} = 0.004$. This process was executed for all contaminated patches each hour that the room was not empty. The amount of LS transferred to an adjacent patch was modeled by applying an assumed transfer coefficient (β~Uniform[0.0,0.05]) to the original level of LS (CFU) on the contaminated patch.

The probability that LS is transported to adjacent patches via (visible) water (p_w) per hour was assumed to be uniformly distributed within the range from 0.01 to 0.05 (p_w~Uniform[0.01, 0.05]). Similar to traffic transmission, the amount of LS added to adjacent patches was calculated by applying a transfer coefficient to the original level of LS (CFU) on the contaminated patch and then evenly distributing the contamination to all adjacent floor patches containing visible water. The values of β and p_w above were assumed as no data were available and their importance was evaluated in the sensitivity analysis.

3.2 Transmission among agents

Transmission onto and between equipment occurred based on connectivity with links, as previously described. The probability of contact (p_{ij}) between agents *i* and *j* was generalized based on their zone categories (Table S1). Similarly, the probability of LS transferred given contact between agents *i* and *j* (τ_{ij}, the transfer coefficient) was also generalized by zone and assumed to be independent of the initial number of bacteria on the surface. No bacteria were lost during transfer events, thus conserving the overall mass. Within the surface population to be transferred, each bacterium was set to act independently such that the overall transfer was the result of a sum of independent Bernoulli trials, and modeled as a Binomial distribution, with the

number of trials equal to the surface microbial population (CFU) and the probability of success equal to the transfer coefficient[12,13]. As supported by these references, this model setup matches the common practice in the modeling community.

3.3 Transmission between agents and patches

Transmission between the equipment agents and environment patches occurred via co-location, where co-location was defined as the presence of an agent (or agents) at different heights on the same patch coordinates. The two mechanisms modeled were: (i) condensation falling from the ceiling to either an agent or the floor below per hour, which was assumed to occur with probability p_c~Uniform[0.01,0.05] and (ii) food falling from a Zone 1 equipment to a floor patch below during production (p_f~Uniform[0.20,0.40], observed by the food safety manager in the processing room). The values of p_c were assumed as no data were available, however their parameter values were intentionally set to wide ranges to test their importance in the sensitivity analysis. The model considered only these two modes of transmission which seemed to be the most relevant to LS transmission between agents and patches.

4. Removal sub-process

In addition to contamination removal by food products being processed, LS was removed from equipment and the environment due to mechanical elimination and disinfection during routine cleaning and sanitation. It was assumed that all bacteria cells on the same surface underwent the same cleaning and sanitation independently, that the overall reduction was immediate, and the resulting population was the sum of independent Bernoulli trials, and modeled as a Binomial distribution, with the number of trials equal to the surface microbial population (CFU) and the probability of success equal to ten raised to the expected daily log reduction, η_d [12,13]. The expected log reduction is sampled each day from a Pert distribution (η_d

~Pert[-8, -6,-1.5, 4])[13]. Therefore, this assumption matches the practices accepted in the modeling community. The model is capable of modeling the presence of sanitizer in the production environment at both lethal and inhibitory concentrations, with the cutoff and importance dependent upon each sanitizer and the frequency with which its concentration is monitored. In the model, areas containing inhibiting concentrations of sanitizer included areas around doorways, where door-foamers or powders may be used, and around drains, where pooling of sanitizer occurs after sanitation. Powdered quaternary ammonia (1.25 g/25 cm^2), commonly used around doorways in ready-to-eat food production facilities, has been previously shown to have inhibitory effect on microbial growth but no significant reduction on LM[15]. Therefore, as in dry areas, in areas of inhibitory sanitizer levels, LS was not able to grow but could survive.

4.1 Cleanability of the equipment and environment

It is well-understood that certain food processing equipment is unable to be effectively cleaned due to its design or the presence of holes or cracks, meaning even when detergent and sanitizer are applied *Listeria,* if present, will remain (along with water and organic matter, in so called niches or harborage sites)[16–18]. Often, hard-to-clean (referred to as "uncleanable") equipment is disassembled and cleaned as part of routine cleaning and sanitation or corrective actions following the detection of contamination. Thus, **EnABLe** agents were set to be either cleanable or uncleanable (Table S2) based on the understanding of a site's unique properties and properties of sites that likely represent niches[19]; however, this status can be changed by the user. For cleanable sites, there was still a probability (γ) that cleaning was not properly executed at the end of the shift, resulting in no reduction of LS on the surface. This random probability of changing the cleanable to uncleanable status was set at 0.01 based on the recognition that random

events, such as human error (i.e., no disassembly of equipment on a given day), could result in unsuccessful cleaning of a site. The value for γ was investigated through scenario analysis and showed no differences when comparing baseline model conclusions to model conclusions for γ=0.1 and 0.001.

5. Environmental monitoring (EM) sub-process

During EM sampling, it was assumed that contamination was homogeneously distributed on the agent or patch surface and that the probability of detection of LS was dependent on the concentration on the surface. A 10% chance of false negative was assumed for concentrations between 0-10 CFU/cm^2; a 1% chance of false negative was assumed for concentrations between 10-100 CFU/cm^2; and, it was assumed that the chance of false negative was negligible for concentrations greater than 100 CFU/cm^2. False positives were not considered possible due to detection of LS over LM and the relatively high specificity for advances in industry-utilized detection methods, especially nucleic acid-based methods[20]. These assumptions were based on expert opinion and the chance of a false negative being dependent on concentration was similarly modeled by Gallagher et al.[11]. The sensitivity of the model to the cut-off values for each chance of false negative was evaluated by scenario analysis and was not shown to impact model conclusions. The two schemes for evaluating the chance of false negative assumption were 0-20 CFU/cm^2, 20-200 CFU/cm^2, and ≥200 CFU/cm^2 and 0-5 CFU/cm^2, 5-50 CFU/cm^2, and ≥50 CFU/cm^2 for 10%, 1% and negligible probability of false negative, respectively.

APPENDIX II

Expert elicitation

Five experts from academia (2) and industry (3) with at least two years of experience in EM and *Listeria* in food processing environments were included as participants in an expert

opinion elicitation. Details of the study objectives and questionnaire logistics were shared before participant agreement and all participant identities and affiliations were kept confidential. An example scenario of the cold-smoked salmon slicing room was provided to familiarize all experts with the product/environment context. A single round of the questionnaire (see below) was administered using a web-based survey tool (Qualtrics). Participants were given two weeks to complete the questionnaire and were offered the opportunity to talk with the primary author about any questions or clarifications, as needed. Expert responses (minimum, most likely, maximum, confidence level) were used to define Pert distributions (minimum, most likely, maximum, scaling parameter) for model parameters.

REFERENCES

1. Eklund, M. W. *et al.* Incidence and sources of *Listeria monocytogenes* in cold-smoked fishery products and processing plants. *J. Food Prot.* **58,** 502–508 (1995). doi: 10.4315/0362-028X-58.5.502
2. Hoelzer, K. *et al.* Estimation of *Listeria monocytogenes* transfer coefficients and efficacy of bacterial removal through cleaning and sanitation. *Int. J. Food Microbiol.* **157,** 267–277 (2012). doi: 10.1016/j.ijfoodmicro.2012.05.019
3. Wen, J., Karthikeyan, S., Hawkins, J., Anantheswaran, R. C. & Knabel, S. J. *Listeria monocytogenes* responds to cell density as it transitions to the long-term-survival phase. *Int. J. Food Microbiol.* **165,** 326–331 (2013). doi: 10.1016/j.ijfoodmicro.2013.05.014
4. Giménez, B. & Dalgaard, P. Modelling and predicting the simultaneous growth of *Listeria monocytogenes* and spoilage micro-organisms in cold-smoked salmon. *J. Appl. Microbiol.* **96,** 96–109 (2004). doi: 10.1046/j.1365-2672.2003.02137.x

5. Begot, C., Lebert, I. & Lebert, A. Variability of the response of 66 *Listeria monocytogenes* and *Listeria innocua* strains to different growth conditions. *Food Microbiol.* **14,** 403–412 (1997). doi: 10.1006/fmic.1997.0097
6. Petran, R. L. & Zottola, E. A. A study of factors affecting growth and recovery of *Listeria monocytogenes* Scott A. *J. Food Sci.* **54,** 458–460 (1989). doi: 10.1111/j.1365-2621.1989.tb03105.x
7. FDA/FSIS. *Quantitative Assessment of Relative Risk to Public Health from Foodborne Listeria monocytogenes Among Selected Categories of Ready-to-Eat Foods*. (Food and Drug Administration, United States Department of Agriculture, 2003). Available at: https://www.fda.gov/downloads/food/foodscienceresearch/ucm197330.pdf. (Accessed: 26th June 2018).
8. Vogel, B. F., Hansen, L. T., Mordhorst, H. & Gram, L. The survival of *Listeria monocytogenes* during long term desiccation is facilitated by sodium chloride and organic material. *Int. J. Food Microbiol.* **140,** 192–200 (2010). doi: 10.1016/j.ijfoodmicro.2010.03.035
9. Hansen, L. T. & Vogel, B. F. Desiccation of adhering and biofilm *Listeria monocytogenes* on stainless steel: Survival and transfer to salmon products. *Int. J. Food Microbiol.* **146,** 88–93 (2011). doi: 10.1016/j.ijfoodmicro.2011.01.032
10. Kastbjerg, V. G. & Gram, L. Model systems allowing quantification of sensitivity to disinfectants and comparison of disinfectant susceptibility of persistent and presumed nonpersistent *Listeria monocytogenes*. *J. Appl. Microbiol.* **106**, 1667–1681 (2009). doi: 10.1111/j.1365-2672.2008.04134.x

11. Gallagher, D. L., Ebel, E. D. & Kause, J. R. *FSIS Risk Assessment for Listeria monocytogenes in Deli Meats*. (United States Department of Agriculture, Food Safety and Inspection Service, 2003). Available at: https://www.fsis.usda.gov/shared/PDF/Lm_Deli_Risk_Assess_Final_2003.pdf. (Accessed on: 25th June 2018).
12. Tenenhaus-Aziza, F., Daudin, J. J., Maffre, A. & Sanaa, M. Risk-based approach for microbiological food safety management in the dairy industry: the case of *Listeria monocytogenes* in soft cheese made from pasteurized milk. *Risk Anal.* **34,** 56–74 (2014). doi: 10.1111/risa.12074
13. FDA/FSIS. *Interagency Risk Assessment: Listeria monocytogenes in Retail Delicatessens; Technical Report*. 1-175 (Food and Drug Administration, United States Department of Agriculture, 2013). Available at: https://www.fsis.usda.gov/wps/wcm/connect/c0c6dfbc-ad83-47c1-bcb8-8db6583f762b/Lm-Retail-Technical-Report.pdf?MOD=AJPERES. (Accessed on: 26th June 2018).
14. Chambers, M. K., Ford, M. R., White, D. M., Barnes, D. L. & Schiewer, S. Transport of fecal bacteria by boots and vehicle tires in a rural Alaskan community. *J. Environ. Manage.* **90,** 961–966 (2009). doi: 10.1016/j.jenvman.2008.03.008
15. Ceylan, E. *Validation of quaternary ammonia and hydrogen peroxide powder for control of Listeria monocytogenes in ready-to-eat meat and poultry plants*. 1–14 (Silliker, Inc., 2011).
16. Ferreira, V., Wiedmann, M., Teixeira, P. & Stasiewicz, M. J. *Listeria monocytogenes* persistence in food-associated environments: epidemiology, strain characteristics, and implications for public health. *J Food Prot* **77,** 150–170 (2014). doi: 10.4315/0362-028X.JFP-13-150

17. Carpentier, B. & Cerf, O. Review--Persistence of *Listeria monocytogenes* in food industry equipment and premises. *Int. J. Food Microbiol.* **145,** 1–8 (2011). doi: 10.1016/j.ijfoodmicro.2011.01.005

18. Tompkin, R. B. Control of *Listeria monocytogenes* in the food-processing environment. *J. Food Prot.* **65,** 709–725 (2002). doi: 10.4315/0362-028X-65.4.709

19. Simmons, C. K. & Wiedmann, M. Identification and classification of sampling sites for pathogen environmental monitoring programs for *Listeria monocytogenes*: Results from an expert elicitation. *Food Microbiol.* **75,** 2–17 (2018). doi:10.1016/j.fm.2017.07.005

20. Wiedmann, M., Wang, S., Post, L. & Nightingale, K. Assessment criteria and approaches for rapid detection methods to be used in the food industry. *J. Food Prot.* **77,** 670–690 (2014). doi: 10.4315/0362-028X.JFP-13-138

EnABLe parameter expert elicitation via Qualtrics

Start of Block: Objectives

Q1.1 Thank you for agreeing to take part in this expert opinion elicitation.

Your identity will be kept confidential.

Q1.2 We are developing a model to study the behavior of microbial contamination (i.e. *Listeria* spp.) in the food processing environment. Your participation will help to elicit information regarding (1) how ***Listeria* spp. may be introduced to** and remain present in a finished product room of a food processing facility, and (2) the **transfer of *Listeria* spp.** within and between equipment and environment under several scenarios common to food processing.

Q1.3 **Food processing plant scenario:** For the purposes of this expert elicitation, imagine a finished product room of a farm-raised cold smoked salmon processing facility with several processing lines. Within this room, smoked salmon fillets are sliced, portioned and packaged. In this room, there are 10 employees that work a 10 hour shift and process 1000 fillets per shift. All questions pertain to Listeria spp.

Q1.4 **Please provide your name.**

____

____

____

____

End of Block: Objectives

Start of Block: Model variables

Q2.1 What are the three most important environment/equipment attributes that affect the presence, survival and transmission of *Listeria* spp. in the finished product room of the smoked salmon facility?

____

____

____

____

____

End of Block: Model variables

Start of Block: Introduction - probability

Q3.1 The following question refers to scenarios that may introduce *Listeria* into the finished product room. For each scenario, first read the series of events that describe what must subsequently occur to introduce contamination. Then, use the categories in Table 1 to give the probability that each occurs and provide your level of confidence in this answer.

Q3.2 **Objects that move into and out of the room (i.e. trolley, cart, product bins)**
In the event these objects enter into the finished product room, it is possible that the object:

- brings *Listeria* spp. into the room, and
- contacts another item in the finished product room, and
- transfers *Listeria* upon contact with another item

Q3.3 **Employee's hands**
Each time an employee enters the finished product room, it is possible that the employee:

- has *Listeria* on their hands, and
- does not properly wash and disinfect their hands upon entry into the finished product room, and
- contaminates their gloves with their hands

Q3.4 **Employee's work-assigned footwear**

Each time an employee enters the finished product room, it is possible that the employee:

- has *Listeria* on their work-assigned footwear, and
- does not properly scrub or cover footwear

Q3.5 **Food entering the finished product room**

Each time food enters the finished product room to be processed, it is possible that the food item:

- is contaminated with *Listeria*, and
- transfers *Listeria* to the first surface it contacts, and
- transfers *Listeria* to subsequent surfaces it contacts

Q3.6 **Unpredictable event (i.e. roof leak, maintenance, drain backs up)**

During a shift, there may be events that:

- cause interruptions or unplanned stops in production, or
- bring visitors or additional employees into the room, or
- increase the likely presence of *Listeria* in the room

Q3.7 Taking all of these steps into account, for each scenario give the category that describes the probability *Listeria* spp. is introduced into the environment or equipment of the finished product room **during different times** and provide your overall level of confidence

Table 1. Probability categories and interpretations

**Category**	**Interpretation**
Negligible	Event is so rare, that in practical terms in can be ignored
Low	Event is very rare
Moderate	Event occurs sometimes
High	Event occurs often
Almost certain	Event almost always occurs

	Hour prior to production shift	First half of production shift	Second half of production shift	Confidence
Objects that move into and out of the room (i.e. trolley, cart, product bins)	▼	▼	▼	▼
Employee's hands	▼	▼	▼	▼
Employee's work-assigned footwear	▼	▼	▼	▼
Food entering the finished product room	▼	▼	▼	▼
Unpredictable event (i.e. roof leak, maintenance, drain backs up)	▼	▼	▼	▼
Other	▼	▼	▼	▼
Other	▼	▼	▼	▼

End of Block: Introduction - probability

Start of Block: Introduction – Load

Q4.1 *Listeria* spp. may be introduced into the finished product room via the scenarios presented in the previous question. Now, estimate the amount of *Listeria* spp. that may be introduced into the finished product room by a single event. Write in a maximum and minimum concentration, select the most likely **level** from Table 2, and then provide your confidence in these responses.

Table 2. Listeria contamination levels

LEVEL	Range in CFU/ cm^2 (or g)
NEGLIGIBLE	≤ 1
LOW	> 1 and ≤ 10
MODERATE	> 10 and ≤ 1000
HIGH	> 1000 and ≤ 10,000
VERY HIGH	> 10,000

Q4.2

	Maximum Concentration CFU/cm^2 (or CFU/g)	Minimum Concentration CFU/cm^2 (or CFU/g)	Most Likely Level CFU/cm^2 (or CFU/g)	Confidence
	Max	Min		
Objects that move into and out of the room (i.e. trolley, cart, product bins)			▼	▼
Employee's hands			▼	▼
Employee's work-assigned footwear			▼	▼
Food entering the finished product room			▼	▼
Unpredictable event (i.e. roof leak, maintenance, drain backs up)			▼	▼
Other			▼	▼
Other			▼	▼

End of Block: Introduction - Load

Start of Block: Transfer

Q5.1 After introduction to the environment/equipment of the finished product room, *Listeria* can be spread via **direct and indirect modes of contact**. For an indirect contact example, an employee moves product between the conveyor belt and the vacuum packing machine. If the conveyor belt is contaminated, we are interested in the probability that transfer of *Listeria* to the vacuum packing machine occurred within one hour. [Note: The amount of *Listeria* transferred is a separate parameter that has been estimated from published literature.]

For each scenario below, suppose there is a mode of contact (indirect or direct) between ITEM A and ITEM B and that *ITEM A* is contaminated (*Listeria* is evenly distributed on its surface). What is the probability (%) that transfer of ***Listeria* spp. from ITEM A (contaminated) to ITEM B (uncontaminated)** occurs **within an hour of production?** Write the range of probability in the corresponding empty boxes (without 'xx').

Q5.2 At the slicer, Employee 1 feeds product onto the slicer in belt. After it is automatically sliced, Employee 2 takes the product from the slicer out belt and moves it to a conveyor belt. There is a drain below the slicer.
What is the probability with which transfer of *Listeria* spp. from **ITEM A (contaminated) to ITEM B (uncontaminated) occurs within an hour of production**? Write the probability range (i.e. 20-30%) in the corresponding empty boxes (without 'xx').

	B. Slicer in belt (zone 1)	B. Slicer out belt (zone 1)	B. Slicer cover (zone 2)	B. Slicer rollers (zone 2)	B. Slicer foot (zone 3)	B. Drain below (zone 3)	B. Employee 1	B. Employee 2
A. Slicer in belt (zone 1)	xx							
A. Slicer out belt (zone 1)		xx						
A. Slicer cover (zone 2)			xx					
A. Slicer rollers (zone 2)				xx				
A. Slicer foot (zone 3)	xx	xx	xx	xx	xx	xx	xx	xx
A. Drain below (zone 3)	xx	xx	xx	xx	xx	xx	xx	xx
A. Employee 1							xx	
A. Employee 2								xx

Q5.3 At the scaling table, Employee 1 stands on a floor mat at a table with a scale next to the conveyor line. Employee 1 takes product from the conveyor belt, weighs a portion and places the portion back onto the conveyor belt.

What is the probability with which transfer of *Listeria* spp. from ITEM A (contaminated) to ITEM B (uncontaminated) occurs within an hour of production? Write the probability range (i.e. 20-30%) in the corresponding empty boxes (without 'xx').

	B. Scale table top (zone 1)	B. Conveyor belt (zone 1)	B. Scale panel (zone 2)	B. Conveyor framework (zone 2)	B. Floor below (zone 3)	B. Floor mat (zone 3)	B. Employee 1
A. Scale table top (zone 1)	xx						
A. Conveyor belt (zone 1)		xx					
A. Scale panel (zone 2)			xx				
A. Conveyor framework (zone 2)				xx			
A. Floor below (zone 3)	xx	xx	xx	xx	xx	xx	xx
A. Floor mat (zone 3)	xx	xx	xx	xx	xx	xx	xx
A. Employee 1							xx

Q5.4 Employee 1 stands at the vacuum machine. Employee 2 brings product in plastic sleeves to the steel table next to the vacuum machine. Employee 1 picks up product, positions it along the seal bar, vacuum seals it and removes it from the machine.

What is the probability with which transfer of *Listeria* spp. from ITEM A (contaminated) to ITEM

B (uncontaminated) occurs within an hour of production? Write the probability range (i.e. 20-30%) in the corresponding empty boxes (without 'xx').

	B. Steel table top (zone 1)	B. Vacuum seal bar (zone 2)	B. Vacuum machine (zone 2)	B. Vacuum feet (zone 3)	B. Floor below (zone 3)	B. Employee 1	B. Employee 2
A. Steel table top (zone 1)	xx						
A. Vacuum seal bar (zone 2)		xx					
A. Vacuum machine (zone 2)			xx				
A. Vacuum feet (zone 3)	xx	xx	xx	xx	xx	xx	xx
A. Floor below (zone 3)	xx	xx	xx	xx	xx	xx	xx
A. Employee 1						xx	
A. Employee 2							xx

Q20 **This is the end of the survey.**
Click back (<<) to review/change your responses.
Click next (>>) if you are finished and are ready to **SUBMIT** your responses. You will not be able to make changes or access the survey once submitted.

End of Block: Transfer

Supplemental Tables and Figures

Table S1. EnABLe parameters, distribution information, values, and sources for *Listeria* spp. transmission between agents.

Symbol	Description[1]	Distribution	Mean	5th-95th percentile	Reference
p_{11}	Probability of contact from contaminated surface in Zone 1 to another surface in Zone 1	Pert (0.6, 0.65, 0.7, 4)	0.65	[0.62, 0.68]	*expert opinion*
p_{12}	Probability of contact from contaminated Zone 1 to Zone 2	Pert (0.4, 0.53, 0.6, 4)	0.52	[0.46, 0.57]	*expert opinion*
p_{13}	Probability of contact from contaminated Zone 1 to Zone 3	Pert (0.25, 0.3, 0.3, 4)	0.29	[0.27, 0.30]	*expert opinion*
p_{14}	Probability of contact from contaminated Zone 1 to an employee	Pert (0.6, 0.6, 0.8, 4)	0.63	[0.60, 0.70]	*expert opinion*
p_{21}	Probability of contact from contaminated Zone 2 to Zone 1	Pert (0.3, 0.35, 0.4, 4)	0.35	[0.32, 0.38]	*expert opinion*
p_{22}	Probability of contact from contaminated Zone 2 to another Zone 2	Pert (0.2, 0.25, 0.25, 4)	0.24	[0.22, 0.25]	*expert opinion*
p_{23}	Probability of contact from contaminated Zone 2 to Zone 3	Pert (0.2, 0.2, 0.23, 4)	0.20	[0.20, 0.21]	*expert opinion*
p_{24}	Probability of contact from contaminated Zone 2 to an employee	Pert (0.2, 0.25, 0.3, 4)	0.25	[0.22, 0.28]	*expert opinion*
p_{31}	Probability of contact from contaminated Zone 3 to Zone 1	Pert (0.0, 0.01, 0.05, 4)	0.01	[0.003, 0.03]	*assumed* [2]
p_{32}	Probability of contact from contaminated Zone 3 to Zone 2	Pert (0.0, 0.01, 0.05, 4)	0.01	[0.003, 0.03]	*assumed*
p_{33}	Probability of contact from contaminated Zone 3 to another Zone 3	Pert (0.0, 0.01, 0.05, 4)	0.01	[0.003, 0.03]	*assumed*
p_{34}	Probability of contact from contaminated Zone 3 to an employee	Pert (0.0, 0.01, 0.05, 4)	0.01	[0.003, 0.03]	*assumed*
p_{41}	Probability of contact from contaminated employee to Zone 1	Pert (0.75, 0.85, 0.98, 4)	0.85	[0.79, 0.92]	*expert opinion*
p_{42}	Probability of contact from contaminated employee to Zone 2	Pert (0.4, 0.43, 0.45, 4)	0.42	[0.41, 0.44]	*expert opinion*
p_{43}	Probability of contact from contaminated employee to Zone 3	Pert (0.2, 0.23, 0.25, 4)	0.23	[0.21, 0.24]	*expert opinion*
p_{44}	Probability of contact from contaminated employee to another employee	Pert (0.2, 0.23, 0.25, 4)	0.22	[0.21, 0.24]	*expert opinion*
for i = 1, 2, 3 and j = 1, 2, 3 and e=employee:					
τ_{ij}	Probability of *Listeria* spp. transfer from Zone i to Zone j given contact	$10^{Normal\ (-0.28,\ 0.2)}$	0.52	[0.25, 1]	[44, 56]
τ_{ie}	Probability of *Listeria* spp. transfer from Zone i to an employee given contact	$10^{Normal\ (-1.84,\ 0.87)}$	0.01	[0.0005, 0.40]	[44, 56]
τ_{ei}	Probability of *Listeria* spp. transfer from an employee to Zone i given contact	$10^{Normal\ (-1.84,\ 0.87)}$	0.01	[0.0005, 0.40]	[44, 56]
τ_{ee}	Probability of *Listeria* spp. transfer from an employee to another employee given contact	$10^{Normal\ (-3.43,\ 0.79)}$	0.0004	[$2x10^{-5}$, 0.007]	[44, 56]

[1]All parameter values correspond to an hourly time-scale, the time-scale of the model.
[2]Assumptions were made when values were not available from literature or experts. Values of assumed parameters were intentionally set to wide ranges to be able to test their importance in sensitivity analysis.

Table S2. Uncleanable and cleanable agents in the cold-smoked salmon slicing room

**Uncleanable site**	**Hygienic Zone**
mechanical bone separator (MBS) – slicer	1
drums (MBS, skinner)	1
pin-belts	1
pin-bone remover	1
gears and joints	2
roller-sides	2
bettcher knives – holders	2
vacuum machine – seal bar, sides	2
pin-bone remover - motor	2
feet (tables, equipment)	3
wheels (carts, trolleys)	3
floor mats	3
hand sink – wall below	3
condenser units	3
drains	3

**Cleanable site**	**Hygienic Zone**
employees	1
bins (plastic, on trolleys)	1
in/out belts (slicer, skinner, MBS)	1
conveyor belt – top	1
scale table – top	1
steel table – top	1
bettcher knives – blades	1
cutting knife	1
underside (conveyor belt, tables)	2
frames (carts, trolleys)	2
control panels (equipment, scales)	2
scale table – shelf	2
windows	2
switches (bettcher knives, slicers)	2
framework (tables, conveyor belts)	2
steel table – leg	2
slicer – drain	2
slicer – cover	2
slicer rack	3
trash can	3
hoses	3
hand sink – faucet	3
hand sink – basin	3

Table S3. Historical data validation results

LS Prevalence Outcome	Observed[1]	EnABLe Mean	*p*-value
**Beginning Shift**	11.3	4.8	0.03
**Middle Shift**	11.8	14.8	0.51
**End Shift**	12.5	18.3	0.15
**Monday**	8.1	4.0	0.26
**Tuesday**	2.3	8.5	0.17[2]
**Wednesday**	25.6	13.1	0.04
**Thursday**	14.0	16.7	0.62
**Friday**	9.3	20.0	0.05
**Skinning Area**	22.5	18.9	0.69
**Trimming Area**	27.3	13.8	0.08
**Slicer A**	19.0	21.7	0.64
**Slicer B**	5.9	21.3	0.003
**Packing Area**	2.0	2.7	1.0[2]
**FCS overall**	13.9	14.6	0.79
**NFCS overall**	5.0	1.7	0.44

[1]Hu et al., 2006 observed prevalence on fixed sites in smoked-salmon slicing room.
[2]Chi-square approximation may be inaccurate due to low counts, so Fisher's Exact Test is reported.

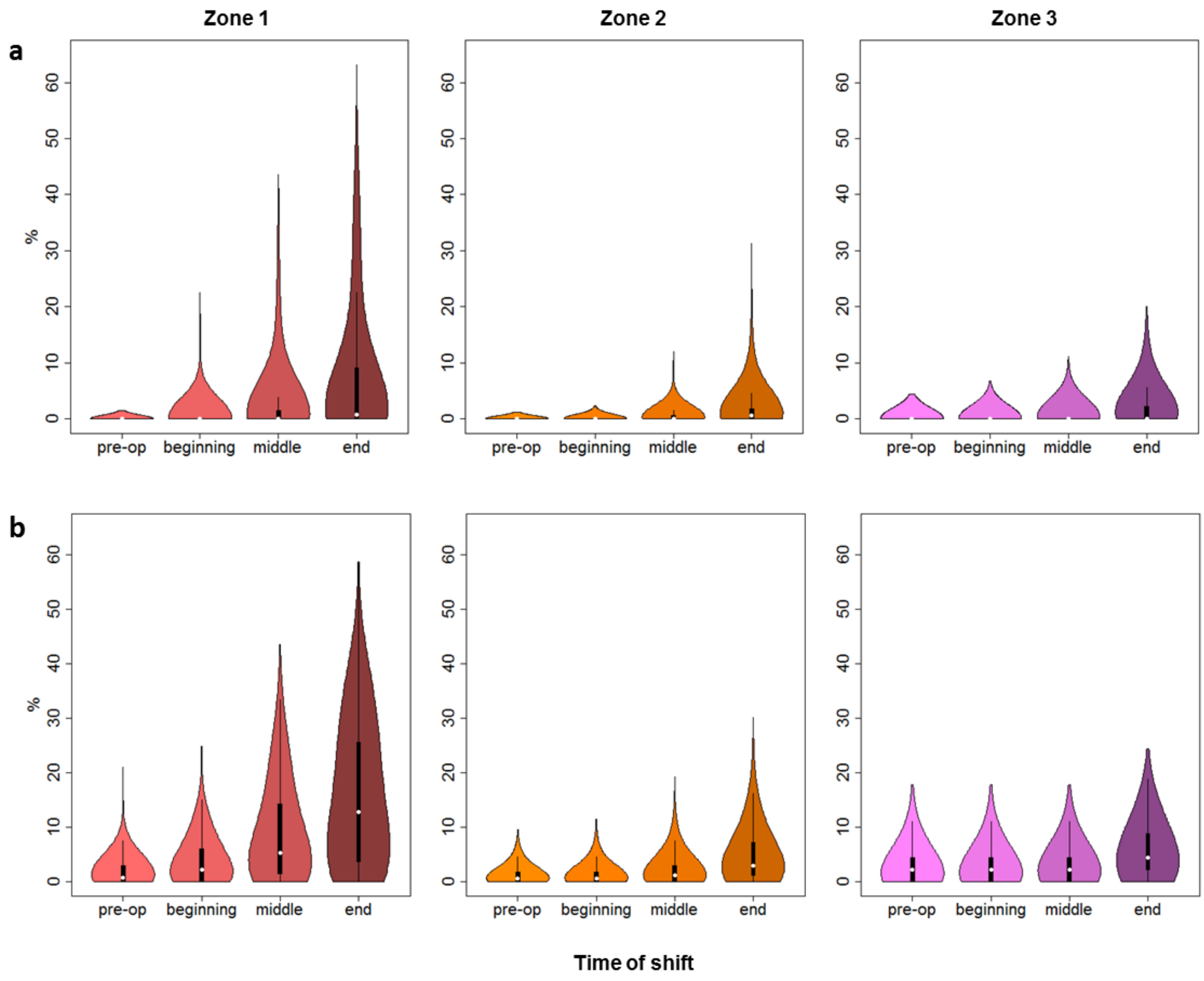

Figure S1. *Listeria* spp. (LS) prevalence on different surface types (characterized by their proximity to food products, with Zone 1 being in contact, and Zone 2 and Zone 3 being non-contact) in the cold-smoked salmon slicing room on two arbitrarily chosen days of the week: **(a)** Monday and **(b)** Wednesday. Results are shown using violin plots, with the central white dot representing the median value, the black bar representing the interquartile range (IQR), the black line representing 95% confidence interval, and the outer shape representing the kernel density plot of all possible values (the thickest section indicates the mode).

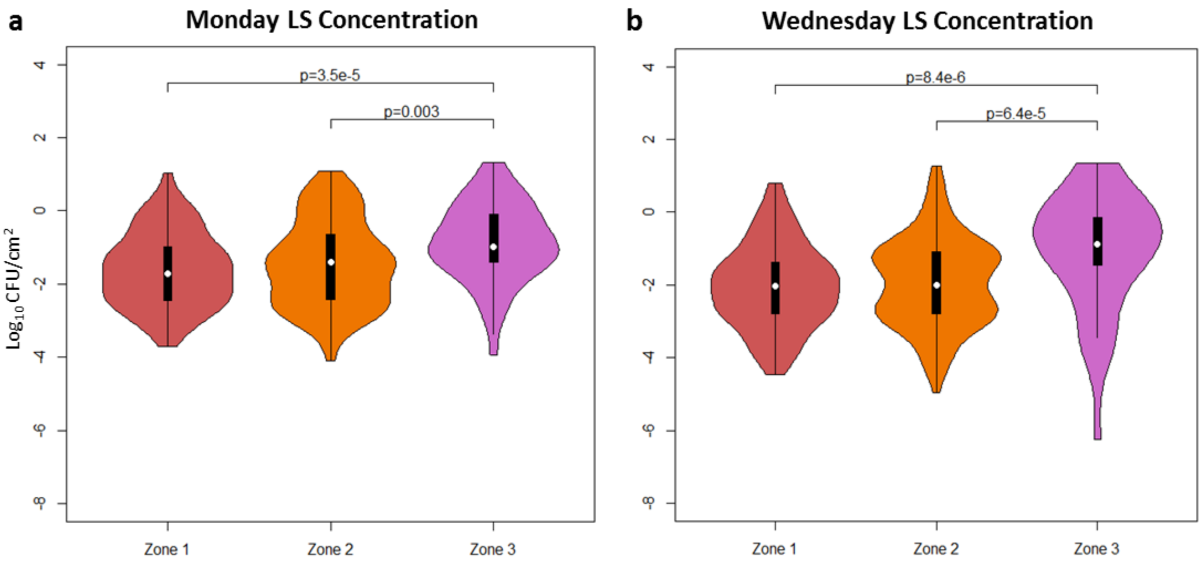

Figure S2. *Listeria* spp. (LS) concentration on different surface types (characterized by their proximity to food products with Zone 1 being in contact, and Zone 2 and Zone 3 being non-contact) on two arbitrarily chosen days of the modeled week, **(a)** Monday and **(b)** Wednesday in the cold-smoked salmon slicing room. Simulation results for the concentration on surfaces (Log_{10} CFU/cm^2), if contaminated at the middle of the shift on Monday, are shown as violin plots, with the central white dot representing the median value, the black bar representing the interquartile range (IQR), the black line representing 95% confidence interval, and the outer shape representing the kernel density plot of all possible values (the thickest section indicates the mode).

Table S4. Agent cluster analysis results according to physical attributes

A-I	A-II	A-III
plastic-cart-wheel	plastic-cart-top	employee
plastic-cart-wheel	plastic-cart-handle	cutting-table-top
drain	plastic-cart-corner	cutting-table-top
drain	plastic-cart-top	cutting-table-top
drain	plastic-cart-handle	slicer-exit-belt
drain	plastic-cart-corner	slicer-in-belt
drain	employee	slicer-pin-belt
drain	employee	slicer-exit-belt
drain	employee	slicer-in-belt
slicer-feet	employee	slicer-pin-belt
slicer-feet	employee	slicer-exit-belt
slicer-feet	employee	slicer-in-belt
slicer-feet	employee	slicer-pin-belt
hand-sink-wallbelow	employee	slicer-exit-belt
hand-sink-faucet	employee	slicer-in-belt
slicer-rack	employee	slicer-pin-belt
slicer-rack	employee	steel-table-top
slicer-rack	employee	steel-table-top
slicer-rack	employee	steel-table-top
trolley-wheel	employee	steel-table-top
trolley-wheel	employee	steel-table-top
trolley-wheel	employee	skinnner-drum
vacuum-feet	employee	skinner-in-belt
vacuum-feet	employee	skinner-out-belt
vacuum-feet	employee	skinnner-drum
hose	employee	skinner-in-belt
hose	employee	skinner-out-belt
hose	employee	bettcher-knife-blade
hose	employee	cutting-knife
trash-can	employee	trolley-bin
trash-can	employee	trolley-bin
floor-mat	employee	plastic-bin
floor-mat	employee	plastic-bin
floor-mat	MBS-belt	vacuum-machine
floor-mat	MBS-drum	vacuum-machine
floor-mat	MBS-feed-channel	scale-table-top
floor-mat	MBS-slicer	conveyor-belt-top
floor-mat	MBS-switch	conveyor-belt-top
floor-mat	MBS-controlpanel	conveyor-belt-top
trolley-wheel	MBS-framework	conveyor-belt-top
	cutting-table-frame	
	cutting-table-top	
	cutting-table-leg	
	cutting-table-underside	
	cutting-table-frame	
	cutting-table-leg	
	cutting-table-underside	
	cutting-table-frame	
	cutting-table-leg	
	cutting-table-underside	
	cutting-table-frame	
	cutting-table-leg	

	cutting-table-underside<br>slicer-controlpanel<br>slicer-on-off-switch<br>slicer-gears-joints<br>slicer-belt-rollersides<br>slicer-cover<br>slicer-drain<br>slicer-controlpanel<br>slicer-on-off-switch<br>slicer-gears-joints<br>slicer-belt-rollersides<br>slicer-cover<br>slicer-drain<br>slicer-controlpanel<br>slicer-on-off-switch<br>slicer-gears-joints<br>slicer-belt-rollersides<br>slicer-cover<br>slicer-drain<br>slicer-controlpanel<br>slicer-on-off-switch<br>slicer-gears-joints<br>slicer-belt-rollersides<br>slicer-cover<br>slicer-drain<br>steel-table-top<br>steel-table-underside<br>steel-table-leg<br>steel-table-leg<br>steel-table-underside<br>steel-table-underside<br>steel-table-leg<br>hand-sink-basin<br>hand-sink-basin<br>conveyor-belt-framework<br>conveyor-belt-top<br>conveyor-belt-rollersides<br>conveyor-belt-underside<br>conveyor-belt-joints<br>conveyor-belt-framework<br>conveyor-belt-top<br>conveyor-belt-rollersides<br>conveyor-belt-underside<br>conveyor-belt-joints<br>conveyor-belt-framework<br>conveyor-belt-top<br>conveyor-belt-rollersides<br>conveyor-belt-underside<br>conveyor-belt-joints<br>conveyor-belt-framework<br>conveyor-belt-top<br>conveyor-belt-rollersides<br>conveyor-belt-underside<br>conveyor-belt-joints<br>skinner-controlpanel<br>skinner-rollersides	

	skinner-window<br>skinner-controlpanel<br>skinner-rollersides<br>skinner-window<br>condenser-unit<br>condenser-unit<br>condenser-unit<br>bettcher-knife-holder<br>bettcher-knife-switch<br>bettcher-knife-hose<br>bettcher-knife-blade<br>bettcher-knife-holder<br>bettcher-knife-switch<br>bettcher-knife-hose<br>cutting-knife<br>cutting-knife<br>cutting-knife<br>pin-bone-remover<br>pin-bone-remover-hose<br>pin-bone-remover-motor<br>trolley-frame<br>trolley-frame<br>trolley-frame<br>trolley-bin<br>vacuum-sealbar<br>vacuum-window<br>vacuum-sealbar<br>vacuum-window<br>vacuum-machine<br>vacuum-sealbar<br>scale-table-top<br>scale-table-scale<br>scale-table-scalepanel<br>scale-table-framework<br>scale-table-shelf<br>scale-table-top<br>scale-table-scale<br>scale-table-scalepanel<br>scale-table-framework<br>scale-table-shelf<br>scale-table-top<br>scale-table-scale<br>scale-table-scalepanel<br>scale-table-framework<br>scale-table-shelf<br>scale-table-top<br>scale-table-scale<br>scale-table-scalepanel<br>scale-table-framework<br>scale-table-shelf<br>scale-table-top<br>scale-table-scale<br>scale-table-scalepanel<br>scale-table-framework<br>scale-table-shelf<br>scale-table-scale	

	scale-table-scalepanel<br>scale-table-framework<br>scale-table-shelf<br>scale-table-top<br>scale-table-scale<br>scale-table-scalepanel<br>scale-table-framework<br>scale-table-shelf<br>scale-table-top<br>scale-table-scale<br>scale-table-scalepanel<br>scale-table-framework<br>scale-table-shelf<br>scale-table-top<br>scale-table-scale<br>scale-table-scalepanel<br>scale-table-framework<br>scale-table-shelf<br>scale-table-top<br>scale-table-scale<br>scale-table-scalepanel<br>scale-table-framework<br>scale-table-shelf<br>scale-table-top<br>scale-table-scale<br>scale-table-scalepanel<br>scale-table-framework<br>scale-table-shelf<br>scale-table-top<br>scale-table-scale<br>scale-table-scalepanel<br>scale-table-framework<br>scale-table-shelf<br>scale-table-top<br>scale-table-scale<br>scale-table-scalepanel<br>scale-table-framework<br>scale-table-shelf<br>scale-table-top<br>scale-table-scale<br>scale-table-scalepanel<br>scale-table-framework<br>scale-table-shelf<br>scale-table-top<br>scale-table-scale<br>scale-table-scalepanel<br>scale-table-framework<br>scale-table-shelf<br>scale-table-top<br>scale-table-scale<br>scale-table-scalepanel<br>scale-table-framework<br>scale-table-shelf<br>scale-table-top<br>scale-table-scale<br>scale-table-scalepanel	

	scale-table-framework<br>scale-table-shelf<br>scale-table-top<br>scale-table-scale<br>scale-table-scalepanel<br>scale-table-framework<br>scale-table-shelf<br>scale-table-top<br>scale-table-scale<br>scale-table-scalepanel<br>scale-table-framework<br>scale-table-shelf<br>scale-table-top<br>scale-table-scale<br>scale-table-scalepanel<br>scale-table-framework<br>scale-table-shelf<br>scale-table-top<br>scale-table-scale<br>scale-table-scalepanel<br>scale-table-framework<br>scale-table-shelf<br>scale-table-top<br>scale-table-scale<br>scale-table-scalepanel<br>scale-table-framework<br>scale-table-shelf<br>scale-table-top<br>scale-table-scale<br>scale-table-scalepanel<br>scale-table-framework<br>scale-table-shelf<br>scale-table-top<br>scale-table-scale<br>scale-table-scalepanel<br>scale-table-framework<br>scale-table-shelf<br>employee<br>conveyor-belt-framework<br>conveyor-belt-top<br>conveyor-belt-framework<br>conveyor-belt-top<br>trolley-frame<br>trolley-bin	

Table S5. Agent cluster analysis results according to contamination outcomes

C-I	C-II	C-III
plastic-cart-wheel	plastic-cart-top	drain
drain	plastic-cart-handle	drain
slicer-gears-joints	plastic-cart-corner	employee
hand-sink-faucet	plastic-cart-top	employee
pin-bone-remover	plastic-cart-handle	employee
vacuum-feet	plastic-cart-corner	employee
scale-table-framework	plastic-cart-wheel	employee
scale-table-framework	drain	employee
	drain	cutting-table-top
	drain	cutting-table-top
	drain	cutting-table-top
	employee	slicer-exit-belt
	employee	slicer-in-belt
	employee	slicer-pin-belt
	employee	slicer-exit-belt
	employee	slicer-in-belt
	employee	slicer-pin-belt
	employee	slicer-exit-belt
	employee	slicer-in-belt
	employee	slicer-pin-belt
	employee	slicer-exit-belt
	employee	slicer-in-belt
	employee	slicer-pin-belt
	employee	skinnner-drum
	employee	skinner-in-belt
	employee	skinner-out-belt
	employee	skinner-window
	employee	skinnner-drum
	employee	skinner-in-belt
	employee	skinner-out-belt
	employee	skinner-window
	employee	bettcher-knife-blade
	employee	cutting-knife
	MBS-belt	trolley-bin
	MBS-drum	trolley-bin
	MBS-feed-channel	conveyor-belt-top
	MBS-slicer	conveyor-belt-top
	MBS-switch	
	MBS-controlpanel	
	MBS-framework	
	cutting-table-frame	
	cutting-table-top	
	cutting-table-leg	
	cutting-table-underside	
	cutting-table-frame	
	cutting-table-leg	
	cutting-table-underside	
	cutting-table-frame	
	cutting-table-leg	
	cutting-table-underside	
	cutting-table-frame	
	cutting-table-leg	

	cutting-table-underside<br>slicer-controlpanel<br>slicer-on-off-switch<br>slicer-gears-joints<br>slicer-feet<br>slicer-belt-rollersides<br>slicer-cover<br>slicer-drain<br>slicer-controlpanel<br>slicer-on-off-switch<br>slicer-gears-joints<br>slicer-feet<br>slicer-belt-rollersides<br>slicer-cover<br>slicer-drain<br>slicer-controlpanel<br>slicer-on-off-switch<br>slicer-feet<br>slicer-belt-rollersides<br>slicer-cover<br>slicer-drain<br>slicer-controlpanel<br>slicer-on-off-switch<br>slicer-gears-joints<br>slicer-feet<br>slicer-belt-rollersides<br>slicer-cover<br>slicer-drain<br>steel-table-top<br>steel-table-top<br>steel-table-underside<br>steel-table-leg<br>steel-table-top<br>steel-table-top<br>steel-table-leg<br>steel-table-underside<br>steel-table-top<br>steel-table-top<br>steel-table-underside<br>steel-table-leg<br>hand-sink-basin<br>hand-sink-basin<br>hand-sink-wallbelow<br>conveyor-belt-framework<br>conveyor-belt-top<br>conveyor-belt-rollersides<br>conveyor-belt-underside<br>conveyor-belt-joints<br>conveyor-belt-framework<br>conveyor-belt-top<br>conveyor-belt-rollersides<br>conveyor-belt-underside<br>conveyor-belt-joints<br>conveyor-belt-framework<br>conveyor-belt-top<br>conveyor-belt-rollersides	

	conveyor-belt-underside<br>conveyor-belt-joints<br>conveyor-belt-framework<br>conveyor-belt-top<br>conveyor-belt-rollersides<br>conveyor-belt-underside<br>conveyor-belt-joints<br>skinner-controlpanel<br>skinner-rollersides<br>skinner-controlpanel<br>skinner-rollersides<br>condenser-unit<br>condenser-unit<br>condenser-unit<br>bettcher-knife-holder<br>bettcher-knife-switch<br>bettcher-knife-hose<br>bettcher-knife-blade<br>bettcher-knife-holder<br>bettcher-knife-switch<br>bettcher-knife-hose<br>cutting-knife<br>cutting-knife<br>cutting-knife<br>pin-bone-remover-hose<br>pin-bone-remover-motor<br>slicer-rack<br>slicer-rack<br>slicer-rack<br>slicer-rack<br>trolley-frame<br>trolley-wheel<br>trolley-frame<br>trolley-wheel<br>trolley-frame<br>trolley-wheel<br>trolley-bin<br>plastic-bin<br>plastic-bin<br>vacuum-machine<br>vacuum-sealbar<br>vacuum-window<br>vacuum-machine<br>vacuum-sealbar<br>vacuum-feet<br>vacuum-window<br>vacuum-machine<br>vacuum-sealbar<br>vacuum-feet<br>scale-table-top<br>scale-table-scale<br>scale-table-scalepanel<br>scale-table-framework<br>scale-table-shelf<br>hose<br>hose	

	hose<br>hose<br>trash-can<br>trash-can<br>floor-mat<br>floor-mat<br>floor-mat<br>floor-mat<br>floor-mat<br>floor-mat<br>floor-mat<br>floor-mat<br>scale-table-top<br>scale-table-scale<br>scale-table-scalepanel<br>scale-table-framework<br>scale-table-shelf<br>scale-table-top<br>scale-table-scale<br>scale-table-scalepanel<br>scale-table-framework<br>scale-table-shelf<br>scale-table-top<br>scale-table-scale<br>scale-table-scalepanel<br>scale-table-framework<br>scale-table-shelf<br>scale-table-top<br>scale-table-scale<br>scale-table-scalepanel<br>scale-table-framework<br>scale-table-shelf<br>scale-table-top<br>scale-table-scale<br>scale-table-scalepanel<br>scale-table-framework<br>scale-table-shelf<br>scale-table-top<br>scale-table-scale<br>scale-table-scalepanel<br>scale-table-framework<br>scale-table-shelf<br>scale-table-top<br>scale-table-scale<br>scale-table-scalepanel<br>scale-table-framework<br>scale-table-shelf<br>scale-table-top<br>scale-table-scale<br>scale-table-scalepanel<br>scale-table-framework<br>scale-table-shelf<br>scale-table-top<br>scale-table-scale<br>scale-table-scalepanel<br>scale-table-framework	

	scale-table-shelf<br>scale-table-top<br>scale-table-scale<br>scale-table-scalepanel<br>scale-table-framework<br>scale-table-shelf<br>scale-table-top<br>scale-table-scale<br>scale-table-scalepanel<br>scale-table-framework<br>scale-table-shelf<br>scale-table-top<br>scale-table-scale<br>scale-table-scalepanel<br>scale-table-framework<br>scale-table-shelf<br>scale-table-top<br>scale-table-scale<br>scale-table-scalepanel<br>scale-table-framework<br>scale-table-shelf<br>scale-table-top<br>scale-table-scale<br>scale-table-scalepanel<br>scale-table-framework<br>scale-table-shelf<br>scale-table-top<br>scale-table-scale<br>scale-table-scalepanel<br>scale-table-framework<br>scale-table-shelf<br>scale-table-top<br>scale-table-scale<br>scale-table-scalepanel<br>scale-table-framework<br>scale-table-shelf<br>scale-table-top<br>scale-table-scale<br>scale-table-scalepanel<br>scale-table-framework<br>scale-table-shelf<br>scale-table-top<br>scale-table-scale<br>scale-table-scalepanel<br>scale-table-shelf<br>scale-table-top<br>scale-table-scale<br>scale-table-scalepanel<br>scale-table-framework<br>scale-table-shelf<br>scale-table-top<br>scale-table-scale<br>scale-table-scalepanel<br>scale-table-framework<br>scale-table-shelf<br>scale-table-top	

	scale-table-scale<br>scale-table-scalepanel<br>scale-table-shelf<br>scale-table-top<br>scale-table-scale<br>scale-table-scalepanel<br>scale-table-framework<br>scale-table-shelf<br>scale-table-top<br>scale-table-scale<br>scale-table-scalepanel<br>scale-table-framework<br>scale-table-shelf<br>employee<br>conveyor-belt-framework<br>conveyor-belt-top<br>conveyor-belt-top<br>conveyor-belt-framework<br>conveyor-belt-top<br>conveyor-belt-top<br>trolley-frame<br>trolley-wheel<br>trolley-bin	
